# Mitochondrial unfolded protein response gene *Clpp* is required to maintain ovarian follicular reserve during aging, for oocyte competence, and development of pre‐implantation embryos

**DOI:** 10.1111/acel.12784

**Published:** 2018-05-30

**Authors:** Tianren Wang, Elnur Babayev, Zongliang Jiang, Guangxin Li, Man Zhang, Ecem Esencan, Tamas Horvath, Emre Seli

**Affiliations:** ^1^ Department of Obstetrics, Gynecology and Reproductive Sciences Yale School of Medicine New Haven Connecticut; ^2^ Department of Obstetrics, Gynecology and Reproductive Center Shengjing Hospital of China Medical University Shenyang China; ^3^ Department of Surgery Yale School of Medicine New Haven Connecticut; ^4^ Department of Comparative Medicine Yale School of Medicine New Haven Connecticut; ^5^ Present address: Department of Obstetrics, Gynecology, and Reproductive Sciences Northwestern University Chicago Illinois; ^6^ Present address: School of Animal Sciences Louisiana State University Agricultural Center Baton Rouge Louisiana

**Keywords:** aging, CLPP, mTOR, mitochondria, mitochondrial unfolded protein response, oocyte, rapamycin

## Abstract

Caseinolytic peptidase P mediates degradation of unfolded mitochondrial proteins and activates mitochondrial unfolded protein response (mtUPR) to maintain protein homeostasis. *Clpp* ^−/−^ female mice generate a lower number of mature oocytes and two‐cell embryos, and no blastocysts. *Clpp* ^−/−^ oocytes have smaller mitochondria, with lower aspect ratio (length/width), and decreased expression of genes that promote fusion. A 4‐fold increase in atretic follicles at 3 months, and reduced number of primordial follicles at 6–12 months are observed in *Clpp* ^−/−^ ovaries. This is associated with upregulation of p‐S6, p‐S6K, p‐4EBP1 and p‐AKT473, p‐mTOR2481 consistent with mTORC1 and mTORC2 activation, respectively, and *Clpp* ^−/−^ oocyte competence is partially rescued by mTOR inhibitor rapamycin. Our findings demonstrate that CLPP is required for oocyte and embryo development and oocyte mitochondrial function and dynamics. Absence of CLPP results in mTOR pathway activation, and accelerated depletion of ovarian follicular reserve.

## 1 INTRODUCTION

In compartmentalized eukaryotic cells, various molecular pathways ensure the integrity of the protein‐folding environments in cytosol, endoplasmic reticulum (ER), and mitochondria. These pathways sense unfolded protein stress in a compartment‐specific manner, and signal to the nucleus for induction of the expression of unfolded protein response (UPR) genes, which are essential for proteostasis (Schinzel & Dillin, 2015). Mitochondrial unfolded protein response (mtUPR) is activated in response to a variety of mitochondrial stress factors including accumulation of unfolded proteins in the mitochondrial matrix (Rath et al., 2012), imbalance between mitochondrial DNA (mtDNA)‐encoded and nuclear‐encoded electron transport chain (ETC) components (Nargund, Pellegrino, Fiorese, Baker & Haynes, 2012; Yoneda et al., 2004), and perturbation of mitochondrial physiology through inhibition of ETC function or accumulation of reactive oxygen species (ROS) (Nargund et al., 2012; Yoneda et al., 2004), and ensures mitochondrial proteostasis by inducing a vigorous transcriptional response that promotes folding, limits import, and reduces translation of mitochondrial proteins (reviewed in Hill & Van Remmen, 2014; Jensen & Jasper, 2014).

Mitochondrial unfolded protein response was first described in *Caenorhabditis elegans*, where mitochondrial stress involving unfolded proteins upregulates the mitochondrial matrix caseinolytic peptidase P (CLPP), which cleaves misfolded proteins (Benedetti, Haynes, Yang, Harding & Ron, 2006; Haynes, Petrova, Benedetti, Yang & Ron, 2007). Cleaved proteins are then exported to cytosol, where they activate the stress activated transcription factor 1 (ATFS1). ATFS1 then enters the nucleus and activates Ubiquitin‐like 5 (UBL5) to form a complex with DVE1 and to induce transcription of mitochondrial chaperones, such as heat shock protein 6 (HSP6) and HSP10 (Haynes, Yang, Blais, Neubert & Ron, 2010; Haynes et al., 2007). In addition, mtUPR induces coenzyme Q biosynthesis, glycolysis, and mitochondrial fission (Aldridge, Horibe & Hoogenraad, 2007), altering mitochondrial metabolism and dynamics to promote mitochondrial function and cell survival during stress. Mitochondrial unfolded protein response and the role of CLPP seem to be conserved in mammals (Benedetti et al., 2006; Zhao et al., 2002), where activation of JNK/c‐JUN pathway leads to the expression of transcription factor C/EBP‐homologous protein (CHOP), which, together with C/EBP, mediates the transcription of mtUPR genes (reviewed in Hill & Van Remmen, 2014). It is important that, in addition to inducing transcription of over 400 genes, mtUPR in yeast, *C. elegans*, and mammals are associated with phosphorylation of eukaryotic initiation factor 2 alpha (eIF2a) by general control nonderepressible 2 (GCN2), resulting in global suppression of translation while mRNAs that contain upstream open reading frames (uORFs) are preferentially translated (Delaney et al., 2013; Rath et al., 2012). Transcriptional activation of mtUPR genes and translational suppression seem to be mediated by two parallel mechanisms, both requiring CLPP (Aldridge et al., 2007; Benedetti et al., 2006; Haynes et al., 2007; Zhao et al., 2002) and reviewed in (Jensen & Jasper, 2014; Schulz & Haynes, 2015). It is important that, recessive *Clpp* mutations have been identified in the human Perrault variant of ovarian failure and sensorineural hearing loss (Jenkinson et al., 2013), and global germline *Clpp* knockout mice display auditory deficits and complete female and male infertility, in addition to reduced pre/postnatal survival and marked ubiquitous growth retardation (Gispert et al., 2013).

Mitochondria structure, shape, number, and mtDNA copy number are tightly controlled during mouse and human oocyte and early embryo development (reviewed in Seli, 2016), and adenosine triphosphate (ATP) content of human oocytes correlates with embryo development and in vitro fertilization (IVF) outcome (Van Blerkom, Davis & Lee, 1995). Mature mouse and human oocytes contain somewhere between 50,000 and 550,000 mtDNA copies, with considerable degree of variability between samples (Pikó & Taylor, 1987; Steuerwald et al., 2000). In an interesting manner, despite drastic changes in mitochondria morphology observed during early pre‐implantation embryo development, total number of mitochondria and mtDNA copy number seem to remain unchanged during cleavage divisions, making the oocyte the primary source of mitochondria for pre‐implantation embryos (Piko & Matsumoto, 1976). Mitochondrial DNA replication resumes around the time of blastocyst formation and is first observed in trophectoderm (TE) cells (reviewed in St John, 2014), consistent with the significant increase in the energy needs of the embryo associated with rapid cell proliferation and implantation (Van Blerkom, 2011). Mitochondrial replication, in turn, starts after implantation (Murakoshi et al., 2013; Pikó & Taylor, 1987). Mitochondrial DNA copy number is higher in aneuploid blastocysts (which contain an abnormal chromosome number) and in euploid blastocysts that fail to implant (Fragouli et al., 2015), suggesting that higher mtDNA copy number reflects embryonic stress and is associated with lower reproductive potential.

In this study, we aimed to uncover the mechanisms leading to female infertility in mice with global germline deletion of *Clpp* (Gispert et al., 2013). We found that *Clpp* knockout (*Clpp* ^*−/−*^) mice generate lower number of mature oocytes and two‐cell embryos and no blastocysts and that these deficiencies in oocyte and embryo development are associated with impaired mitochondrial function and dynamics. *Clpp* ^*−/−*^ mice ovaries showed accelerated depletion of follicular reserve, associated with mechanistic target of rapamycin (mTOR) pathway activation.

## 2 RESULTS

### 2.1 *Clpp* is essential for female fertility, oocyte maturation, and embryo development

Male and female *Clpp* ^+/−^ mice appeared phenotypically normal, and intercrossing of the heterozygous mice produced homozygous *Clpp*‐deficient mice with a normal male‐to‐female ratio. This indicated that the targeted disruption of *Clpp* gene did not cause a significant selective disadvantage with regard to sex. *Clpp*‐deficient female mice (3‐month‐old) were viable; however, they were significantly smaller compared to wild‐type (WT). Their uteri and ovaries were also significantly smaller (*n* = 4 for each genotype; Supporting Information Figure S1). Metabolic status of *Clpp*‐deficient female mice was assessed in 3 to 9 months of age. Serum glucose, cholesterol, and phospholipids showed no significant difference between *Clpp*‐deficient and WT mice at any time point, while triglycerides were significantly lower in 9 months old *Clpp* ^−/−^ mice (*p *<* *0.01; Supporting Information Figure S2). To confirm the reported infertility of *Clpp* ^−/−^ female mice (Gispert et al., 2013), we conducted a continuous mating study using sexually mature female mice (*n* = 5 for each genotype) and WT male mice of proven fertility. After 12 weeks of mating, there were no pregnancies or deliveries observed in *Clpp* ^−/−^ female mice. Wild‐type females exhibited normal fertility.

**Figure 1:**
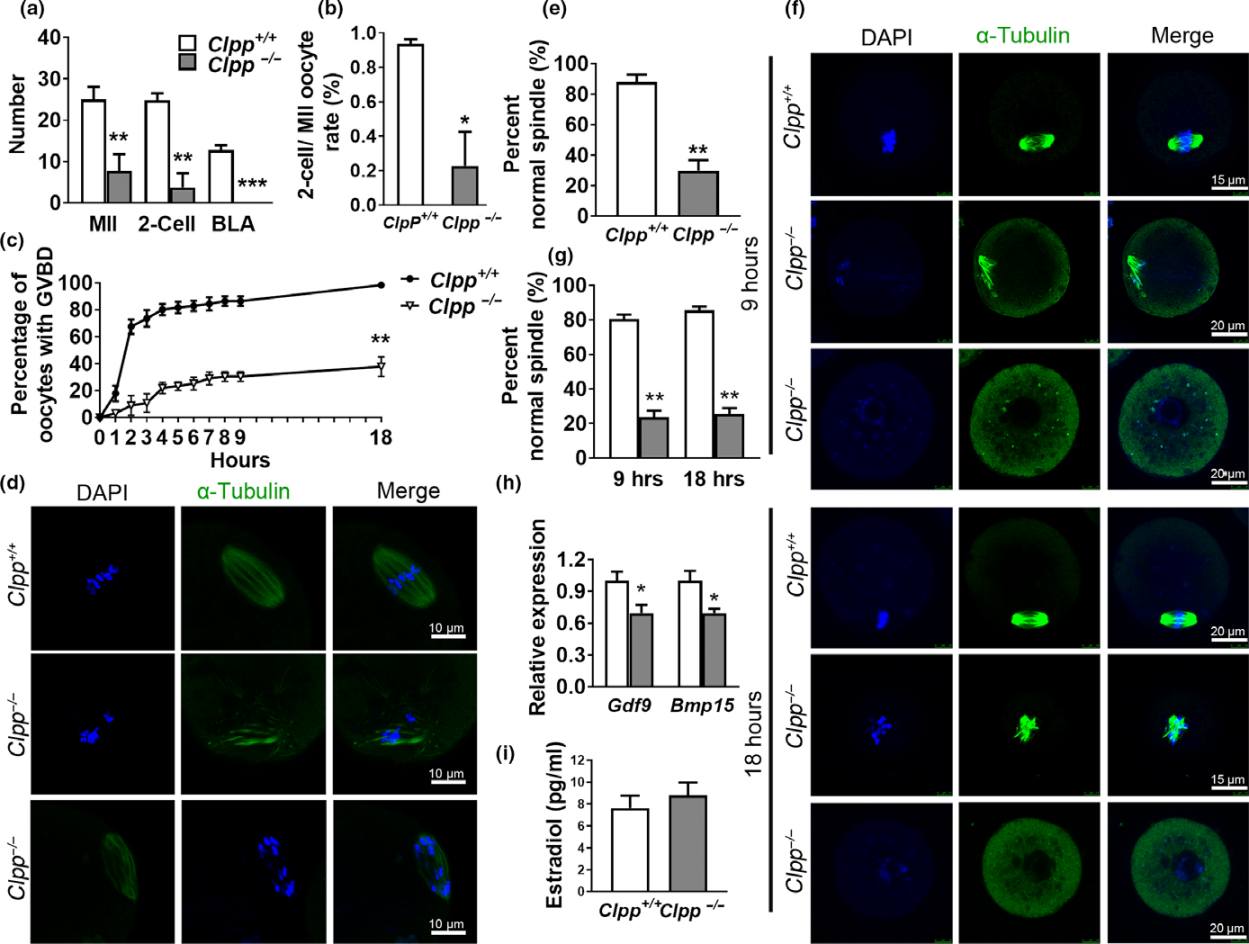
Defective oocyte maturation and embryo development in *Clpp* ^−/−^ female mice. (a) Mature (MII) oocyte, two‐cell embryo, and blastocyst generation by 12‐week‐old *Clpp* ^+/+^ and *Clpp* ^−/−^ female mice. (b) Two‐cell embryo development rate normalized to the number of MII oocytes. (c) GVBD rate of under in vitro maturation (IVM) conditions. (d) Representative spindles from *Clpp* ^+/+^ and *Clpp* ^−/−^ MII oocytes obtained in vivo. Column 1, DAPI (blue); Column 2, anti‐α‐tubulin antibody (green); Column 3, merged images of DAPI and anti‐α‐tubulin. (e) Percentage of in vivo matured *Clpp* ^+/+^ and *Clpp* ^−/−^ MII oocytes with normal chromosome alignment on spindle. (f) Representative spindles from *Clpp* ^+/+^ and *Clpp* ^−/−^ MII oocytes collected at 9 and 18 hr after IVM. (g) Percentage of *Clpp* ^+/+^ and *Clpp* ^−/−^ MI and MII oocytes with normal chromosome alignment on spindle after 9 and 18 hr of IVM. (h) The expression of oocyte‐specific genes *Gdf9* and *Bmp15* assessed using qRT–PCR in *Clpp* ^+/+^ and *Clpp* ^−/−^ GV oocytes. (i) Serum estradiol levels in 12‐week‐old *Clpp* ^+/+^ and *Clpp* ^−/−^ female mice. *p <* *0.05, **p <* *0.01, ***p <* *0.001. The results represent means ± *SEM*. Significance was determined by *t* test. BLA: blastocyst; *Bmp15*: bone morphogenetic protein 15; DAPI: 4′,6‐diamidino‐2‐phenylindole; *Gdf9*: growth differentiation factor 9; GVBD: germinal vesicle breakdown; IVM: in vitro maturation

To determine the cause of female infertility, oocyte and embryo generation were assessed in 3‐month‐old *Clpp* ^+/+^ and *Clpp* ^−/−^ mice (*n* = 4 for each genotype). *Clpp* ^−/−^ mice generated a significantly lower number of mature MII oocytes (7.8 ± 4.0 vs. 25 ± 3.1, *p *<* *0.01) and two‐cell embryos (3.8 ± 3.4 vs. 24.8 ± 1.8, *p *<* *0.01) compared to *Clpp* ^+/+^, and no blastocysts (0 ± 0 vs. 12.8 ± 1.3, *p *<* *0.001) (Figure 1a). The two‐cell embryo development rate was still significantly lower in *Clpp* ^−/−^ mice when the numbers were normalized to MII oocytes in each group (22.7% ± 19.9% vs. 93.7% ± 2.6, *p *<* *0.05; Figure 1b). To characterize the defect in the maturation of *Clpp* ^−/−^ oocytes, in vitro maturation (IVM) was performed and chromatin and spindle morphology were assessed by immunofluorescence. IVM revealed that *Clpp* ^−/−^ GV oocytes had a significantly lower rate of germinal vesicle breakdown (GVBD) after 9 hr (30.5 ± 3.6 vs. 86.5 ± 3.7%, *p *<* *0.001) and 18 hr (37.9 ± 7.2 vs. 98.5 ± 1.5%, *p *<* *0.001; Figure 1c). *Clpp* ^−/−^ mice also had significant lower proportion of normal spindles in MII oocytes in vivo (30 ± 3.4 vs. 88 ± 2.4%, *p *<* *0.01; Figure 1d–e), and MI (23.7 ± 3.8 vs. 80.5 ± 2.6%, *p *<* *0.01) and MII oocytes (25.6 ± 3.6 vs. 85.6 ± 2.2%, *p* < 0.01) in vitro (Figure 1f–g). In addition, the expressions of oocyte‐specific genes *Gdf9* and *Bmp15*, which regulate follicle development, were significantly lower in *Clpp* ^−/−^ oocytes (Figure 1h), while serum estradiol levels were not different after PSMG stimulation (Figure 1i).

### 2.2 Mitochondrial dysfunction is associated with increased ROS and mtDNA, decreased ATP generation, and impaired mitochondrial dynamics and mtUPR pathway gene expression in *Clpp* ^−/−^ oocytes

**Figure 2:**
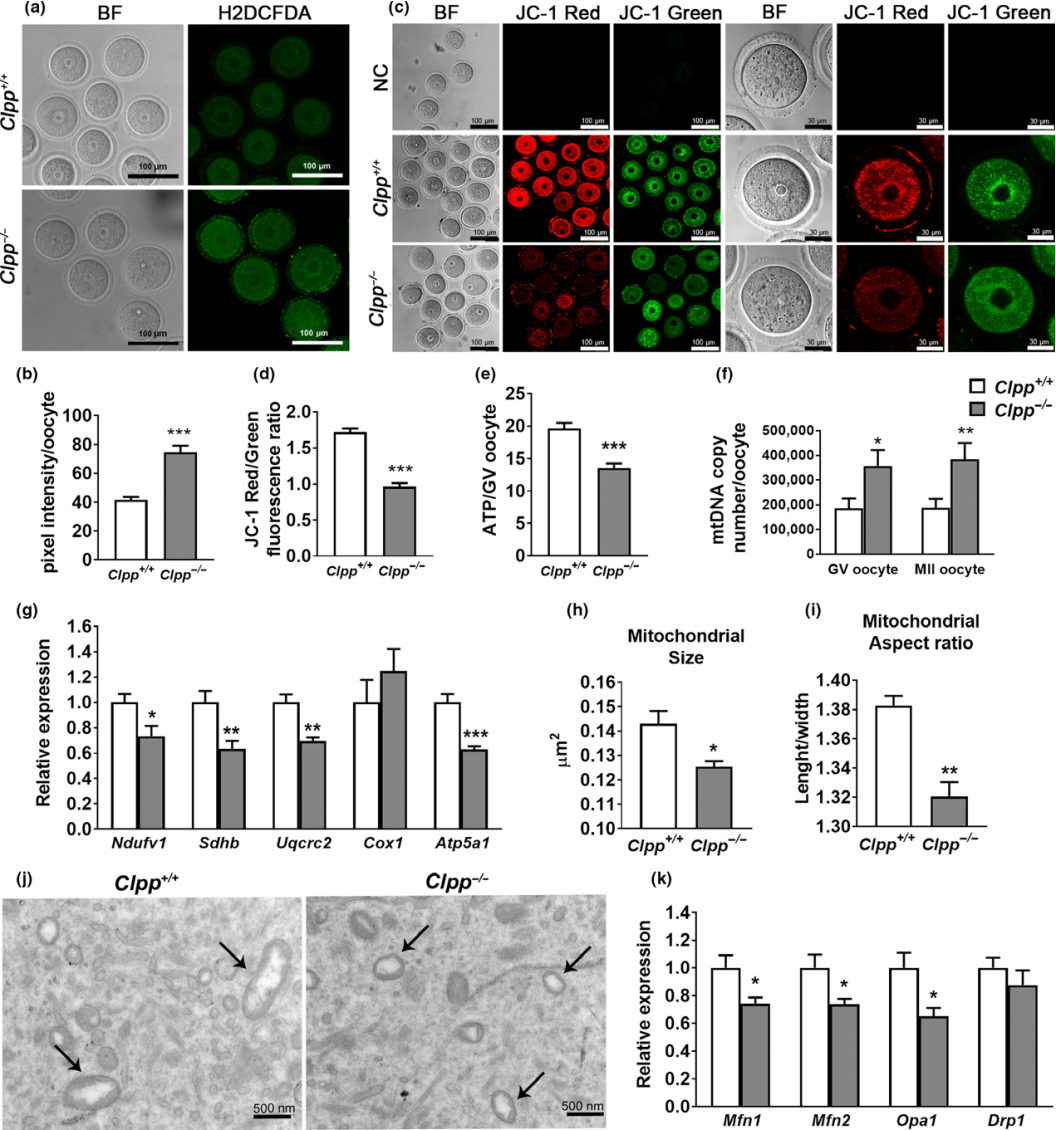
Mitochondrial function and dynamics are impaired in *Clpp* ^−/−^ oocytes. (a) ROS levels in *Clpp* ^+/+^ and *Clpp* ^−/−^ GV oocytes after treatment with H_2_O_2_. (b) Fluorescence pixel intensity of Carboxy‐H_2_ DCFDA was used to measure ROS levels in *Clpp* ^+/+^ and *Clpp* ^−/−^ GV oocytes following H_2_O_2_ treatment. (c) Fluorescent micrographs of GV oocytes stained by mitochondria‐specific probe JC‐1. Red fluorescence represents J‐aggregate and green fluorescence represents JC‐1 monomer. (d) Mitochondrial membrane potential indicated by the red/green fluorescence intensity ratio. (e) ATP level in *Clpp* ^+/+^ and *Clpp* ^−/−^ GV oocytes. (f) mtDNA copy number was determined by qRT–PCR in GV and MII oocytes collected from *Clpp* ^+/+^ and *Clpp* ^−/−^ mice. (g) Expression of respiratory chain genes were assessed using qRT–PCR in GV stage oocytes collected from PMSG‐primed *Clpp* ^+/+^ and *Clpp* ^−/−^ mice. (h) Mitochondrion size (average mitochondrion area) in *Clpp* ^+/+^ and *Clpp* ^−/−^ GV oocytes. (i) Mitochondrion aspect ratio (shape; length/width) in *Clpp* ^+/+^ and *Clpp* ^−/−^ GV oocytes. (j) Representative electronic micrographs of GV oocytes from *Clpp* ^+/+^and *Clpp* ^−/−^ mice. Arrows show mitochondria. (k) The expression of mitochondrial fusion genes *Mfn1, Mfn2, and Opa1* and mitochondrial fission gene *Drp1*. All the oocytes were collected from 12‐week‐old *Clpp* ^+/+^and *Clpp* ^−/−^ mice. *p <* *0.05, **p <* *0.01, ***p <* *0.001. The results represent mean ± *SEM*. Significance was determined by *t* test. Abbreviations: ATP: adenosine triphosphate; *Atp5a1*: ATP synthase, H+ transporting, mitochondrial F1 complex, alpha subunit 1; BF: bright field; carboxy‐H_2_ DCFDA: 6‐carboxy‐2_, 7_‐dichlorodihydrofluorescein diacetate; *Cox1*: cytochrome c oxidase subunit I; *Drp1:* dynamin‐related protein 1; H_2_O_2_: hydrogen peroxide; *Mfn1*: mitofusin 1; *Mfn2*: mitofusin 1; NC: negative control; *Ndufv1*: NADH dehydrogenase (ubiquinone) flavoprotein 1; *Opa1*: mitochondrial dynamin‐like GTPase; ROS: reactive oxygen species; *Sdhb*: succinate dehydrogenase complex iron sulfur subunit B; *Uqcrc2*: ubiquinol cytochrome c reductase core protein 2

Next we compared the mitochondrial function of *Clpp* ^−/−^ oocytes to WT. *Clpp* ^−/−^ GV stage oocytes had higher ROS levels (74.6 ± 4.6 vs. 41.4 ± 2.2 pixel intensity, *p *<* *0.001; Figure 2a–b), and significantly lower mitochondrial membrane potential (0.97 ± 0.05 vs. 1.72 ± 0.05, *p *<* *0.001; Figure 2c–d), and lower ATP levels (13.51 ± 0.7 vs. 19.63 ± 0.9, *p *<* *0.001; Figure 2e). We also measured mtDNA copy number in individual GV and MII oocytes by qPCR, as a marker for mitochondrial distress. Both GV (357,810 ± 63,670 vs. 186,733 ± 38,463, *p *<* *0.05) and MII (384,105 ± 66,531 vs. 188,154 ± 35,496, *p *<* *0.01) stage *Clpp* ^−/−^ oocytes had significantly higher mtDNA copy number compared to WT (Figure 2f). These changes were associated with suppressed expression of subunit I (*Ndufv1*), II (*Sdhb*), III (*Uqcrc2*), and V (*Atp5a1*) of respiratory chain complex genes in *Clpp* ^−/−^ oocytes (Figure 2g). The expression of UPR^mt^ pathway genes, *Hspd1*,* Hspe1*, and *Dnaja3*, were also significantly lower in *Clpp* ^−/−^ oocytes (Supporting Information Figure S5).

EM showed that *Clpp* ^−/−^ oocyte mitochondria were smaller in size (0.125 ± 0.002vs 0.143 ± 0.005 μm^2^, *p *<* *0.05) and had a smaller aspect ratio (length/width; 1.32 ± 0.01 vs. 1.38 ± 0.007; *p *<* *0.01) with a more round contour (Figure 2h–j). This was associated with a decreased expression of *Mfn1, Mfn2*, and *Opa1* (fusion genes) without a change in *Drp1* (fission gene; Figure 2k). In total, these data suggest that mitochondrial function and dynamics are severely affected in *Clpp* ^−/−^ oocytes.

### 2.3 Targeted deletion of *Clpp* results in accelerated depletion of ovarian follicular reserve

**Figure 3:**
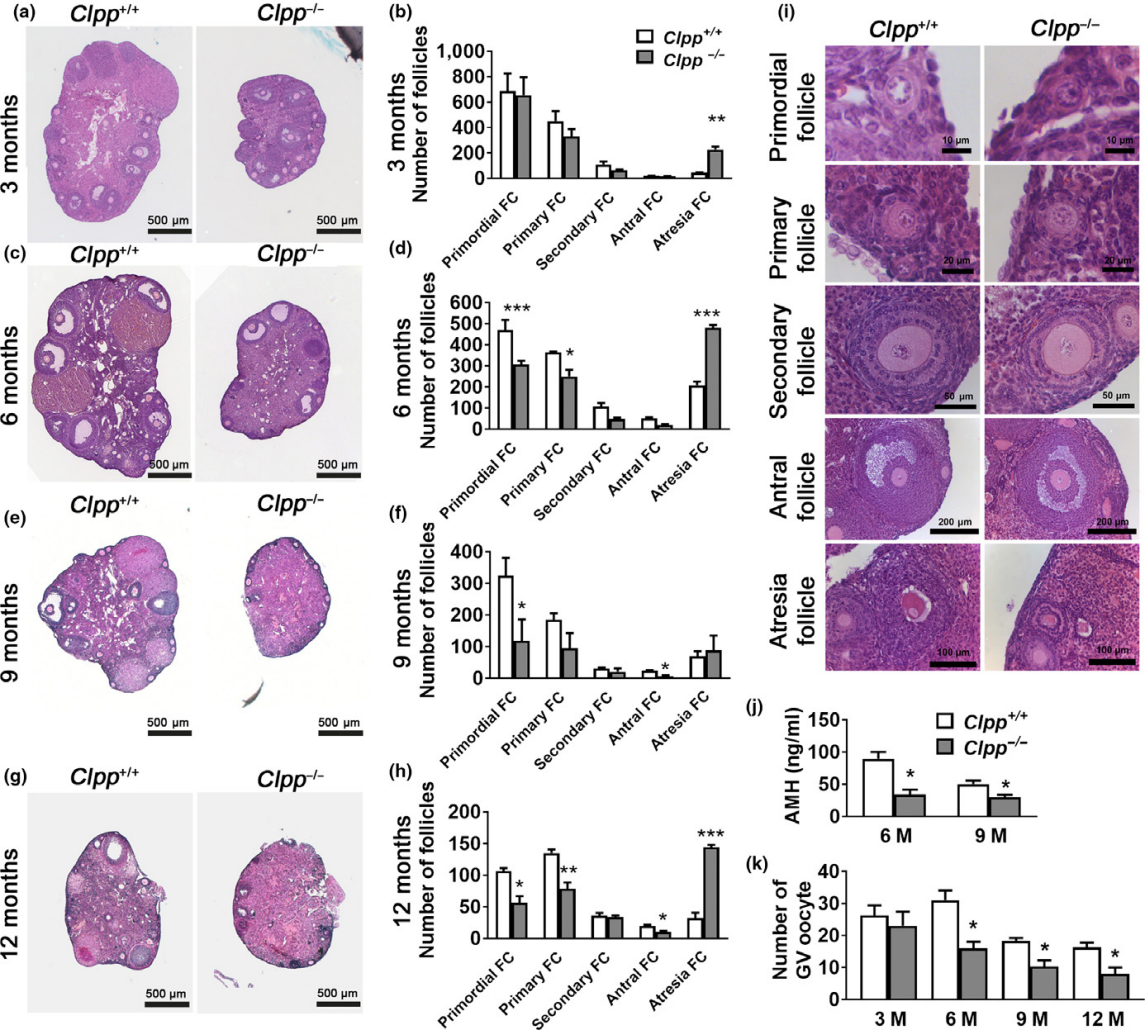
Follicle atresia is accelerated in *Clpp* ^−/−^ mice. (a, c, e, g) Follicle development was assessed in ovaries of unstimulated 3‐, 6‐, 9‐, and 12‐month‐old *Clpp* ^+/+^ and *Clpp* ^−/−^ mice. (b, d, f, h) Follicle counts using ovaries from four mice for each genotype and time point. (i) Representative high‐magnification micrographs of follicles from 3‐month‐old *Clpp* ^+/+^ and *Clpp* ^−/−^ mice at different developmental stages. (j) Serum AMH levels for 6‐ and 9‐month‐old *Clpp* ^+/+^ and *Clpp* ^−/−^ mice. (k) Number of GV oocyte collected from PMSG‐primed *Clpp* ^+/+^ and *Clpp* ^−/−^ mice at 3, 6, 9, and 12 months. Data represent means ± *SEM*. *p <* *0.05, **p <* *0.01, ***p <* *0.001. Significance was determined by *t* test. Abbreviations: AMH: anti‐Müllerian hormone

We assessed follicle development in the ovaries of unstimulated *Clpp* ^+/+^ and *Clpp* ^−/−^ mice at 3, 6, 9, and 12 months of age. At 3 months, the number of primordial (which represent ovarian follicular reserve), primary, secondary, and antral follicles did not differ between *Clpp* ^+/+^ and *Clpp* ^−/−^, while *Clpp* ^−/−^ ovaries had a 4‐fold higher number of atretic follicles (*n* = 4 mice for each genotype; Figure 3a–b). By 6 months, *Clpp* ^−/−^ mice ovaries had significantly lower number of primordial and primary follicles (*n* = 4 mice for each genotype), in addition to higher number of atretic follicles (Figure 3c–d). By 9 months, *Clpp* ^−/−^ ovaries showed a 3‐fold decrease in primordial follicles (*n* = 3 mice for each genotype) (Figure 3e–f), and at 12 months (Figure 3g–h), the number of primordial follicles in *Clpp* ^−/−^ ovaries was approximately half of that seen in *Clpp* ^+/+^(*n* = 3 mice for each genotype). Representative figures of different stages of follicles are shown for 3‐month‐old *Clpp* ^+/+^ and *Clpp* ^−/−^ mice (Figure 3i). Similar results were obtained with follicle density comparisons (Supporting Information Figure S3). Serum anti‐Müllerian hormone (AMH) level was also significantly lower in 6‐ (33.86 ± 7.84 ng/ml vs. 89.60 ± 10.71 ng/ml, *p *<* *0.05) and 9‐ (29.74 ± 3.80 ng/ml vs. 50.00 ± 6.00 ng/ml*, p *<* *0.05) month‐old *Clpp* ^−/−^ mice (Figure 3j). There was no difference between 3‐month‐old *Clpp* ^+/+^ and *Clpp* ^−/−^ mice in the number of GV stage oocytes obtained. However, 6‐, 9‐, and 12‐month‐old *Clpp* ^−/−^ mice generated a significantly lower number of GV oocytes compared to *Clpp* ^+/+^ (*n* = 3 mice for each genotype and for each time point; Figure 3k).

**Figure 4:**
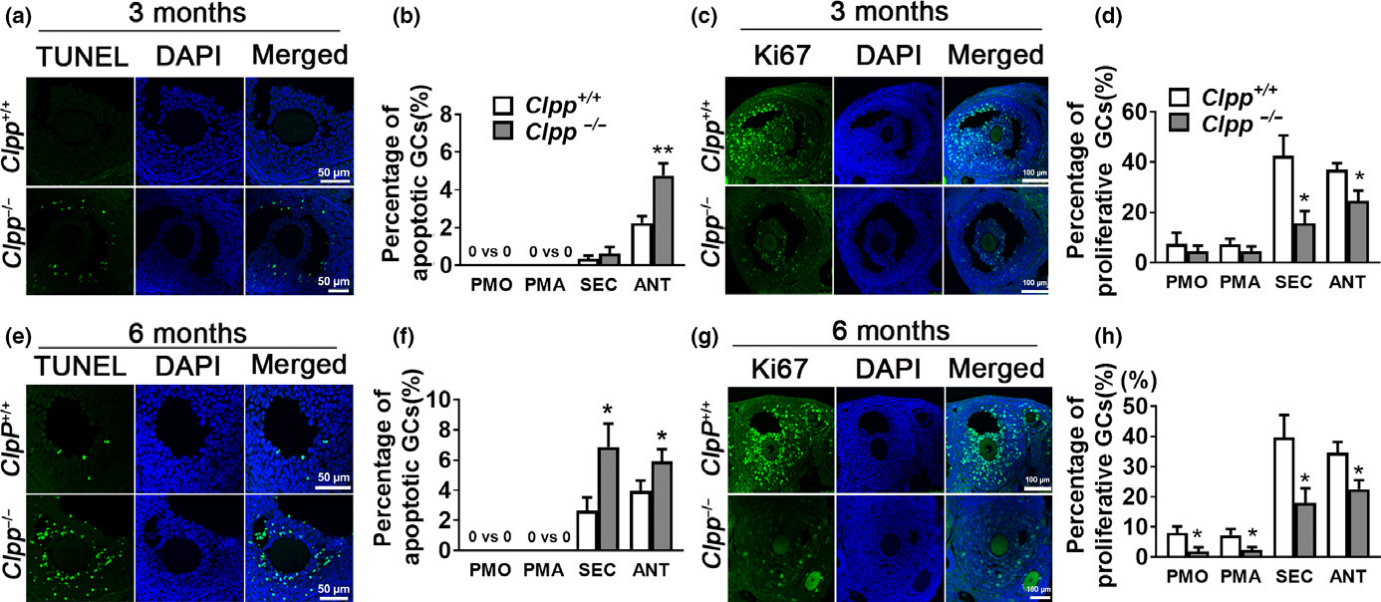
Apoptotic rate is increased and proliferative rate is decreased in *Clpp* ^−/−^ mice granulosa cells. (a and e) Representative micrographs of TUNEL staining in antral follicles of *Clpp* ^*+/+*^ and *Clpp* ^−/−^ mice. (b and f) Percentage of TUNEL positive granulosa cells in different follicular stages of 3‐ and 6‐month‐old *Clpp* ^*+/+*^ and *Clpp* ^−/−^ mice. (c and g) Representative micrographs of Ki67 staining in antral follicles of *Clpp* ^*+/+*^ and *Clpp* ^−/−^ mice. (d and h) Percentage of Ki67‐positive granulosa cells in different follicular stages of 3‐ and 6‐month‐old *Clpp* ^*+/+*^ and *Clpp* ^−/−^ mice. Data represent means ± *SEM*. *p <* *0.05, **p <* *0.01, ***p <* *0.001. Significance was determined by *t* test. Abbreviations: AMH: anti‐Müllerian hormone; ANT: antral follicle; DAPI: 4′,6‐diamidino‐2‐phenylindole; PMA: primary follicle; PMO: primordial follicle; SEC: secondary follicle; TUNEL: terminal deoxynucleotidyl transferase dUTP nick end labeling

Apoptosis and proliferation of granulosa cells at different stage of folliculogenesis were assessed by TUNEL and Ki67 immunofluorescent staining, respectively, in 3‐ and 6‐month‐old *Clpp* ^+/+^ and *Clpp* ^−/−^ mice ovaries. Apoptotic rate of granulosa cells was significantly higher at antral follicle stage in 3‐month‐old *Clpp* ^−/−^ mice (Figure 4a–b) and at secondary and antral follicle stages in 6‐month‐old *Clpp* ^−/−^ mice (Figure 4e–f). Proliferative rate of granulosa cells was significantly lower at both secondary and antral follicle stages in 3‐month‐old *Clpp* ^−/−^ mice (Figure 4c–d) and at all follicle stages from primordial to antral in 6‐month‐old *Clpp* ^−/−^ mice (Figure 4g–h; *p *<* *0.05) Representative micrographs demonstrating granulosa cells positive for TUNEL or Ki67 at primordial, primary, and secondary follicles in 3‐ and 6‐month‐old *Clpp* ^+/+^ and *Clpp* ^−/−^ mice are shown as Supporting Information Figures S6 and S7.

### 2.4 Gene expression is altered in *Clpp* ^−/−^ oocytes

**Figure 5:**
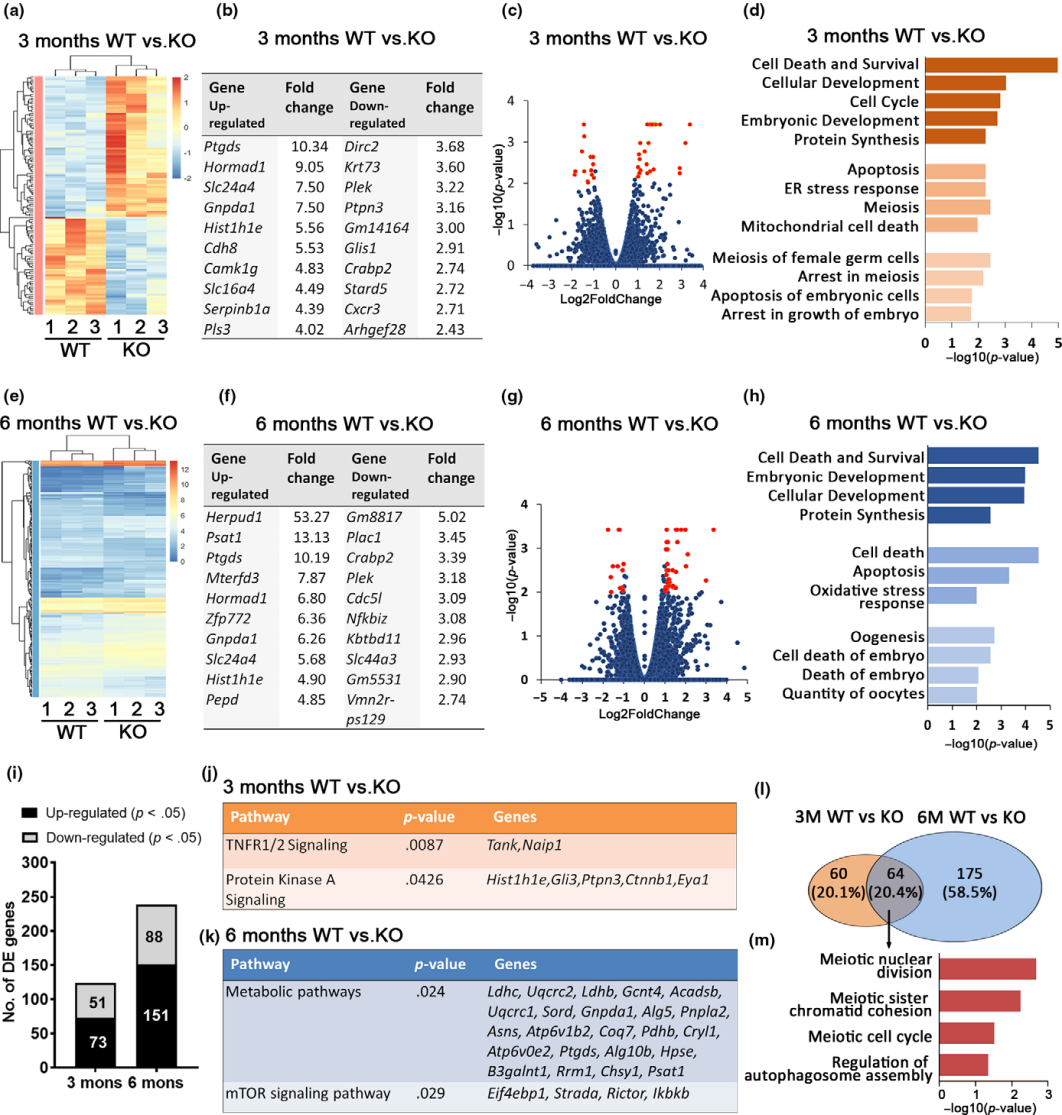
Gene expression is altered in *Clpp* ^−/−^ oocytes. (a, e) Heatmap illustration showing genes differentially expressed between *Clpp* ^−/−^ and *Clpp* ^+/+^ GV stage oocytes from 3‐ and 6‐month‐old mice. The color spectrum ranging from red to blue indicates normalized levels of gene expression from high to low. (b, f) Top 10 upregulated and downregulated annotated genes in *Clpp* ^−/−^ GV oocytes compared to *Clpp* ^+/+^ at 3 and 6 months. (c, g) Volcano plots for RNAseq at 3 and 6 months comparing *Clpp* ^−/−^ and *Clpp* ^+/+^ GV oocytes. Red spot represents −log10 (*p*‐value) ≥ 2; blue spot represents the −log10 (*p*‐value) < 2. (d, h) GO cluster enrichment at 3 and 6 months between *Clpp* ^−/−^ and *Clpp* ^+/+^ GV oocytes. (i) Number of genes that are downregulated or upregulated in 3 and 6 months *Clpp* ^−/−^ GV oocytes compared to *Clpp* ^+/+^. (j, k) Pathway enrichment in 3‐ and 6‐month‐old *Clpp* ^−/−^ mice GV oocytes compared to *Clpp* ^+/+^. (l) Venn diagram of differentially regulated genes at 3 and 6 months between *Clpp* ^−/−^ and *Clpp* ^+/+^ GV oocytes cross‐section showing the overlap. (m) GO cluster enrichment of genes that are differentially expressed in *Clpp* ^−/−^ compared to *Clpp* ^+/+^ GV oocytes in both 3‐ and 6‐month‐old mice. DE: differentially expression; ER: endoplasmic reticulum; GO: gene ontology; KO: knockout; WT: wild‐type

To delineate the gene pathways affected by the absence of *Clpp*, a comprehensive genomewide transcriptomic investigation was conducted. Unsupervised hierarchical clustering of the differentially expressed genes partitioned into two distinct clusters to separate *Clpp* ^−/−^ and *Clpp* ^+/+^ GV oocytes from 3‐month‐old mice (Figure 5a). A total of 124 genes were significantly differentially expressed (*p *<* *0.05) in *Clpp* ^−/−^ oocytes compared to WT (73 upregulated and 51 downregulated; Figure 5c,i, Supporting Information Table S2); top 10 upregulated and downregulated annotated genes in 3‐month‐old *Clpp* ^−/−^ mice oocytes are listed (Figure 5b). Gene ontology (GO) cluster analysis indicated significant over‐representation of elements involved in regulation of cell death, development, meiosis, and embryonic development (Figure 5d). To note, TNFR1/2 signaling pathway and Protein Kinase A signaling pathway were affected in *Clpp* ^−/−^ oocytes (Figure 5j).

Hierarchical clustering of the differentially expressed genes also partitioned into two distinct clusters to separate *Clpp* ^−/−^ and *Clpp* ^+/+^ GV oocytes from 6‐month‐old mice (Figure 5e). A total of 239 genes were significantly differentially expressed in *Clpp* ^−/−^ oocytes compared to WT (151 upregulated and 88 downregulated; Figure 5g,i, Supporting Information Table S3), top 10 upregulated and downregulated annotated genes in *Clpp* ^−/−^ oocytes 6 months are listed (Figure 5f). Gene ontology cluster analysis indicated significant over‐representation of elements involved in regulation of apoptosis, oxidative stress, and oocyte and embryonic development (Figure 5h). In addition, metabolic pathways and aging‐related mTOR signaling pathway were affected in 6 months *Clpp* ^−/−^ oocytes, compared to *Clpp* ^+/+^ oocytes (Figure 5k).

There were 64 differentially expressed genes that overlapped between 3 and 6 months group comparing *Clpp* ^−/−^ to WT, GO cluster of which indicated significant over‐representation of elements involved in meiosis and autophagosome assembly (Figure 5l–m). The biological processes significantly represented among differentially expressed genes included mitochondrial function (*Mrps31* and *Trit1*), cell senescence (*Gpx6, Lamtor1* and *Hist1h1e*), cell growth (*Hormad1* and *Hormad2*), infertility (*Sycp3*), and embryo development (*Gli3*). Differential expression of these genes was confirmed by qRT–PCR (*p *<* *0.05; Supporting Information Figure S4).

### 2.5 mTOR signaling is activated in 6 months *Clpp*‐deficient ovaries

**Figure 6:**
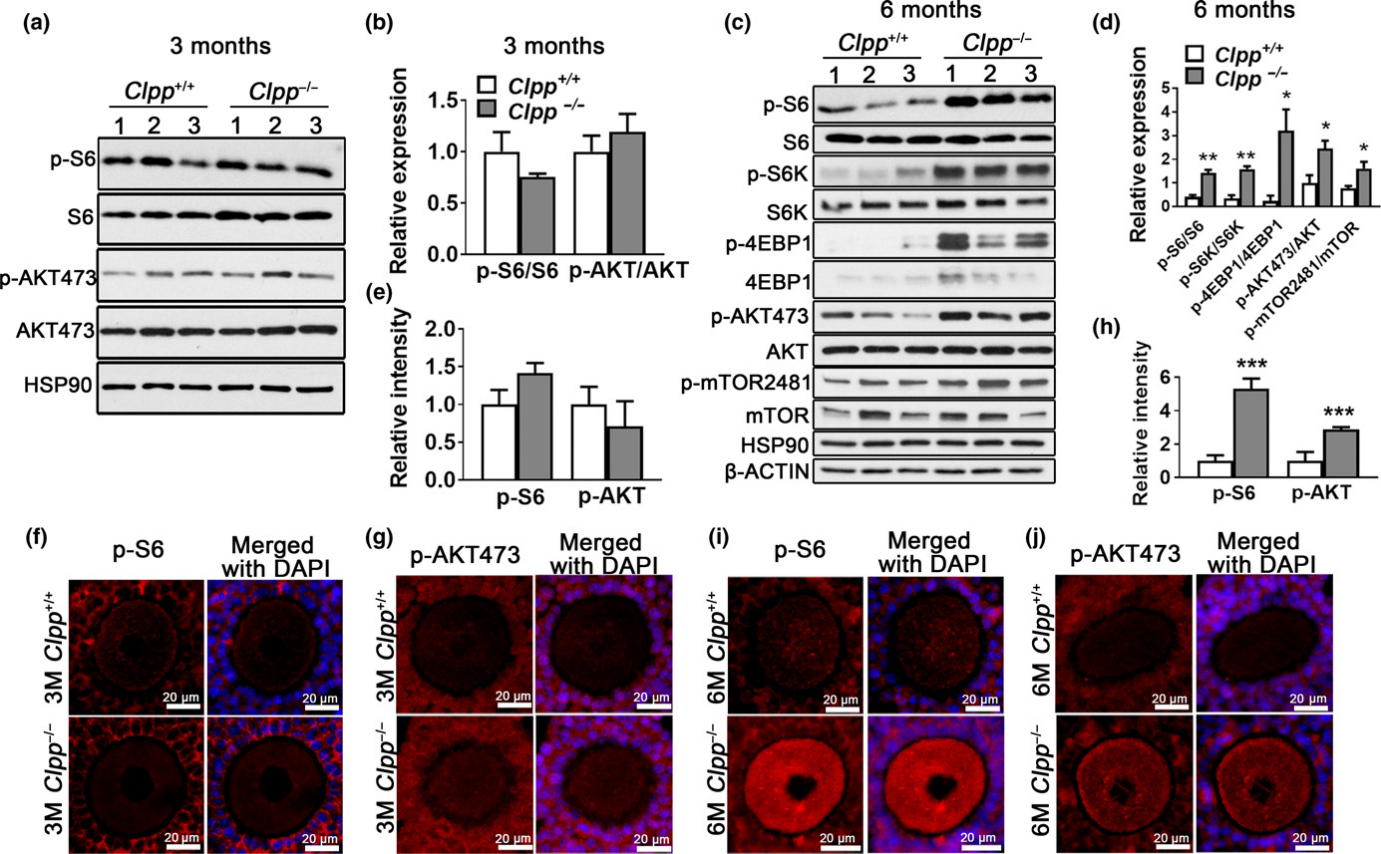
Mechanistic target of rapamycin (mTOR) signaling is activated in *Clpp* ^−/−^ oocytes. (a and b) Western blot analysis for phosphorylated S6 (pS6), total S6, pAKT473, and total AKT in 3‐ month‐old *Clpp* ^+/+^and *Clpp* ^−/−^ mouse ovaries. Band densities were normalized to corresponding S6 and AKT. HSP90 was used as a loading control. (c and d) Western blot analysis for pS6, total S6, pS6K, total S6K, p4EBP1, total 4EBP1, pAKT473, total AKT, p‐mTOR2481, and total mTOR2481 in 6‐month‐old *Clpp* ^+/+^and *Clpp* ^−/−^ mouse ovaries. Band densities were normalized to corresponding S6, S6K, 4EBP1, AKT, and mTOR2481. HSP90 and β‐ACTIN were used as loading controls. (e) Bar chart showing p‐S6 and p‐AKT immunofluorescence intensity in oocytes of 3‐month‐old *Clpp* ^+/+^and *Clpp* ^−/−^ mice. (f and g) Representative micrographs of pS6 and pAKT473 immunofluorescence in 3‐month‐old *Clpp* ^+/+^and *Clpp* ^−/−^ mouse oocytes. (h) Bar chart showing pS6 and pAKT immunofluorescence intensity in oocytes of 6‐month‐old *Clpp* ^+/+^and *Clpp* ^−/−^ mice. (i and j) Representative micrographs of pS6 and pAKT immunofluorescence in 6‐month‐old *Clpp* ^+/+^and *Clpp* ^−/−^ mouse oocytes. Data represent means ± *SEM*. *p <* *0.05. **p <* *0.01. ***p <* *0.001. Significance was determined by *t* test [Correction added on 6 June 2018, after first online publication: Figure 6 has been corrected in this current version.]

As a regulator of aging, and a signaling pathway identified as being altered in 6‐month‐old *Clpp* ^−/−^ oocytes, we next assessed mTOR downstream regulation in *Clpp* ^−/−^ mice ovaries and oocytes compared to WT. Ovaries (*n* = 3 for each genotype) were collected from 3‐ and 6‐month‐old *Clpp* ^+/+^ and *Clpp* ^−/−^ mice and western analysis was performed to assess the expression of p‐S6 and p‐AKT473 proteins, as downstream mediators of mTORC1 and mTORC2 activation (Sarbassov, Guertin, Ali & Sabatini, 2005). There was no significant difference in p‐S6 or p‐AKT473 expression at 3 months between *Clpp* ^−/−^ and *Clpp* ^+/+^ mice ovaries (Figure 6a–b), while both p‐S6 (1 ± 0.30 vs. 3.29 ± 0.56, *p *<* *0.01) and p‐AKT473 (1 ± 0.40 vs. 2.452 ± 0.57 *p *<* *0.05) protein expressions were significantly increased in *Clpp* ^−/−^ mice ovaries at 6 months (Figure 6c–d). Similar to that, immunofluorescence staining showed no significant difference in p‐S6 and p‐AKT473 expression between *Clpp* ^−/−^ and *Clpp* ^+/+^ GV oocytes at 3 months (Figure 6e–g), while both p‐S6 and p‐AKT473 expressions were higher in *Clpp* ^−/−^ GV oocytes at 6 months (*p *<* *0.001; Figure 6h–j), indicating that *Clpp* deletion results in the activation of mTOR pathway. We then tested three additional proteins that mediate mTOR activity, p‐4EBP1 and p‐S6K for mTORC1 (Zoncu, Efeyan & Sabatini, 2011), and p‐mTOR2481 for mTORC2 (Copp, Manning & Hunter, 2009). The expression of these three more proteins was also significantly increased in 6‐month‐old *Clpp* ^−/−^ mice ovaries (Figure 6c–d).

### 2.6 Competence of *Clpp* ^−/−^ oocytes is partially rescued by mTOR inhibitor rapamycin

**Figure 7:**
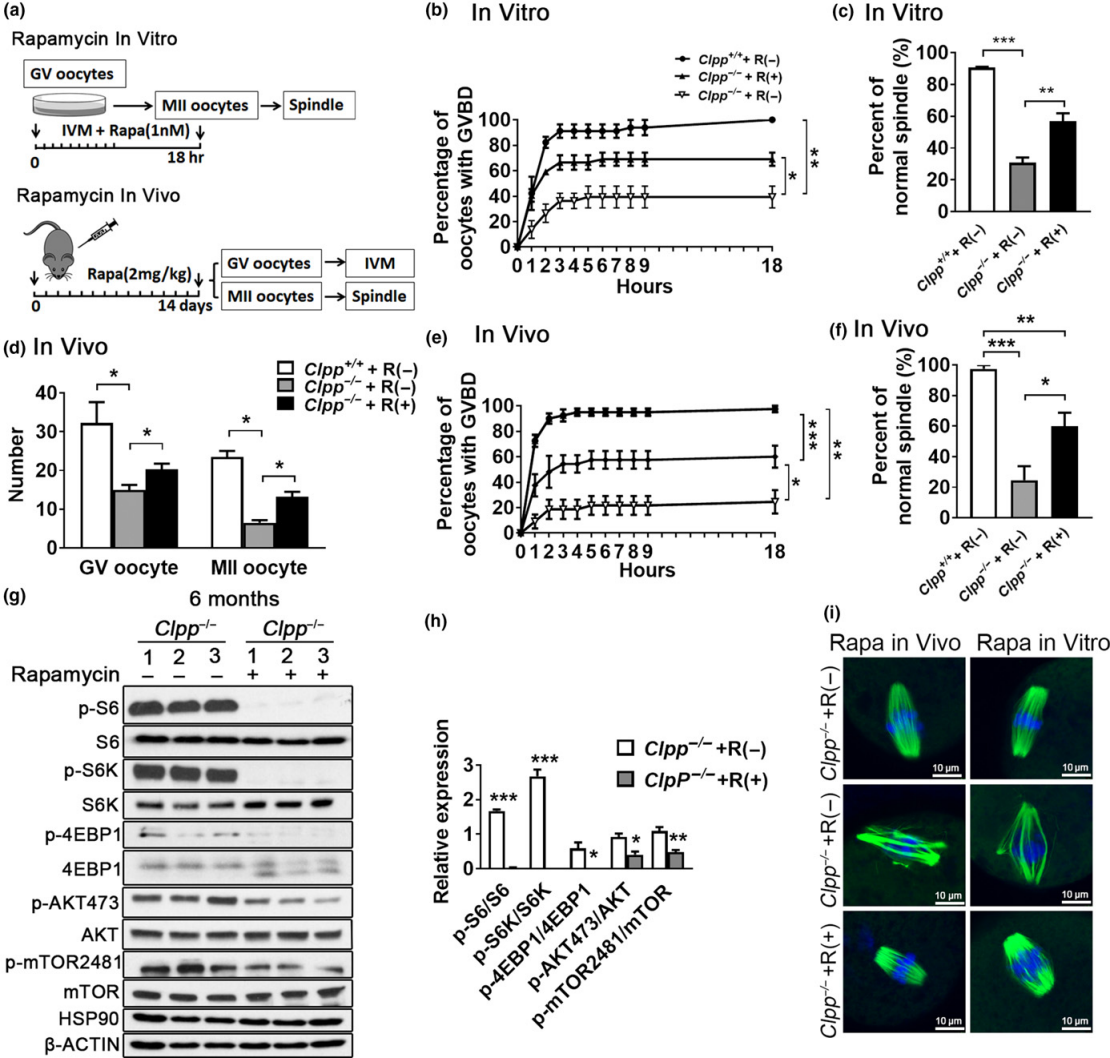
mTOR inhibitor rapamycin partially rescues *Clpp* ^−/−^ oocyte competence. (a) Schematic diagram showing the experimental design in vivo and in vitro. (b and e) GVBD rate of GV oocytes from 6‐month‐old *Clpp* ^+/+^ without rapamycin treatment (*Clpp* ^+/+^ + R[−]); *Clpp* ^−/−^ without rapamycin treatment (*Clpp* ^−/−^ + R[−]); and *Clpp* ^−/−^ with rapamycin treatment (*Clpp* ^−/−^ + R[+]); in vitro and in vivo. (c and f) The percentage of MII oocytes with normal chromosome alignment on spindle in 6‐month‐old *Clpp* ^+/+^ + R(−); *Clpp* ^−/−^ + R(−); *Clpp* ^−/−^ + R(+), in vitro an in vivo. (d) Number of GV and MII oocytes obtained from 6‐month‐old *Clpp* ^+/+^ + R(−); *Clpp* ^−/−^ + R(−); *Clpp* ^−/−^ + R(+), in vivo. (g, h) Western blot analysis for pS6, total S6, pS6K, total S6K, p4EBP1, total 4EBP1, pAKT473, total AKT, p‐mTOR2481, and total mTOR2481 in 6‐month‐old *Clpp* ^−/−^ + R(−) and *Clpp* ^−/−^ + R(+) ovaries in vivo. Band densities were normalized to corresponding S6, S6K, 4EBP1, AKT, and mTOR2481. HSP90 and β‐ACTIN were used as loading controls. (I) Representative spindle morphology from 6‐month‐old *Clpp* ^+/+^ + R(−); *Clpp* ^−/−^ + R(−); and *Clpp* ^−/−^ + R(+) oocytes in vitro and in vivo. Blue fluorescence represents DAPI and green fluorescence represents anti‐α‐tubulin antibody. *p <* *0.05, **p <* *0.01, ***p <* *0.001. The results represent means ± *SEM*. Significance was determined by *t* test and one‐way ANOVA test. GVBD: germinal vesicle breakdown; GV: germinal vesicle; IVM: in vitro maturation; R: rapamycin

After observing that mTOR pathways are activated in *Clpp* ^−/−^ ovaries and oocytes, we tested whether mTOR inhibitor rapamycin could rescue oocyte function. Rapamycin treatment was given both in vitro and in vivo (Figure 7a). For in vitro rescue, 1 nM of rapamycin was added to IVM culture medium and GV oocytes were divided into three groups: *Clpp* ^+/+^ GV oocytes without rapamycin treatment (*Clpp* ^+/+^ + R[−]), *Clpp* ^−/−^ GV oocytes without rapamycin treatment (*Clpp* ^−/−^ + R[−]), and *Clpp* ^−/−^ GV oocytes with rapamycin treatment (*Clpp* ^−/−^ + R[+]). The GVBD rate of *Clpp* ^*−*/−^ + R(+) was significantly improved compared to *Clpp* ^−/−^ + R(−) (69 ± 5.13% vs. 39.33 ± 8.54%, *p *<* *0.05) after 18 hr IVM (Figure 7b). The normal spindle rate was also significantly improved in *Clpp* ^−/−^ + R(+) compared to *Clpp* ^−/−^ + R(−) group (56.84 ± 8.73% vs. 30.71 ± 5.68%, *p *<* *0.01; Figure 7c).

For in vivo rescue, 2 mg/kg of rapamycin was injected intraperitoneally up to 14 days prior to oocyte collection. The three groups were set up as *Clpp* ^+/+^ + R(−), *Clpp* ^−/−^ + R(−), and *Clpp* ^−/−^ + R(+). Both GV (20.3 ± 3 vs. 15 ± 2.6, *p *<* *0.05) and MII oocyte (13.3 ± 2.5 vs. 6.5 ± 1.3, *p *<* *0.05) numbers were significantly increased in *Clpp* ^−/−^ + R(+) compared to *Clpp* ^−/−^ + R(−) group (Figure 7d). In addition, the GVBD rate of *Clpp* ^*−*/−^ + R(+) was significantly improved compared to *Clpp* ^−/−^ + R(−) (60 ± 8.7% vs. 24.5 ± 9.2%, *p *<* *0.05) after 18 hr of IVM, while it was still lower than the *Clpp* ^+/+^ + R(−) group (97.5 ± 2.5%, *p *<* *0.001; Figure 7e). The normal spindle rate was also significantly improved in *Clpp* ^−/−^ + R(+) compared to *Clpp* ^−/−^ + R(−) group (60 ± 17.4% vs. 24.5 ± 18.4%, *p *<* *0.05), but remained lower than *Clpp* ^+/+^ + R(−) group (97.5 ± 5%, *p *<* *0.01) (Figure 7f). The expression of mTOR pathway downstream proteins p‐S6, p‐S6K, p‐4EBP1, p‐AKT473, and p‐mTOR2481 was all significantly inhibited after rapamycin treatment in *Clpp* ^−/−^ + R(+), compared to the *Clpp* ^−/−^ + R(−) group (Figure 7g–h). Representative figures of spindles from each group are shown in Figure 7i.

## 3 DISCUSSION

Gispert et al. observed reduced number of granulosa cell layers and higher amount of apoptotic bodies in *Clpp* ^−/−^ mice follicles and suggested that CLPP deficiency could lead to a selective impairment of granulosa cell differentiation, resulting in female infertility observed in *Clpp* ^−/−^ mice. In the current study, we aimed to first investigate whether CLPP deficiency affects oocyte function and early embryo development. We found *Clpp* ^−/−^ mice to produce a lower number of MII oocytes. In addition, following controlled ovarian hyperstimulation and mating, *Clpp* ^−/−^ mice generated a lower number of two‐cell embryos, and no blastocysts (Figure 1a–b). To further delineate the mechanism of failed pre‐implantation development, we assessed oocyte maturation in vivo and in vitro and observed chromosome misalignment at first and second meiotic metaphases (Figure 1c–g). In total, these findings demonstrate that both oogenesis and pre‐implantation embryo development are impaired in the absence of CLPP.

After establishing that CLPP is required for oocyte and early embryo development, we assessed how CLPP deficiency affects mitochondrial function and dynamics in *Clpp* ^−/−^ mice oocytes. We first focused on parameters reflecting the efficiency of oocyte energy metabolism and found *Clpp* ^−/−^ mice oocytes to have higher amount of ROS, with decreased membrane potential and ATP production (Figure 2a–e), as well as lower expression of genes coding for ETC proteins (Figure 2g). Similar to that observed in human embryos that are aneuploid or fail to implant (Fragouli et al., 2015), *Clpp* ^−/−^ mice oocytes had significantly higher mtDNA copy number (Figure 2f). We then assessed whether the environment with impaired energy metabolism affects mitochondrial dynamics in *Clpp* ^−/−^ oocytes. EM analysis revealed *Clpp* ^−/−^ mice oocytes to have smaller and shorter mitochondria, suggesting decreased fusion (Figure 2h–j). We also found transcripts coding for fusion proteins (*Mfn1*,* Mfn2*,* Opa1*) to be downregulated, while the expression of *Drp*1, which mediates fission, was unchanged (Figure 2k). Overall, both cellular energy metabolism and mitochondrial dynamics seem to be severely affected in *Clpp* ^−/−^ mice oocytes.

Histomorphometric assessment of ovaries from 3‐, 6‐, 9‐, and 12‐month‐old *Clpp* ^−/−^ and WT mice revealed a phenotype consistent with accelerated follicular depletion (Figure 3a–i). *Clpp* ^−/−^ ovaries had a 4‐fold increase in atretic follicles by 3 months, and the number of atretic follicles remained higher at 12 months. Consistent with increased follicular atresia, primordial follicle numbers were lower in *Clpp* ^−/−^ ovaries at 6, 9, and 12 months. Similar to that, primary follicles were also decreased by 6 and 12 months. We also assessed the number of GV stage oocytes that were obtained from *Clpp* ^−/−^ ovaries compared to WT. As predicted by increased atresia and decreased follicle count, *Clpp* ^−/−^ ovaries generated a significantly lower number of GV stage oocytes at 6, 9, and 12 months (Figure 3k). Taken together with chromosome misalignment on spindle at MI and MII stages (Figure 1d–g), and failed blastocyst development (Figure 1a), the increased follicular atresia and decreased number of primordial and primary follicles in *Clpp* ^−/−^ ovaries constitute a phenotype resembling human ovarian aging, which is characterized by follicular depletion, increased aneuploidy and decreased blastocyst development.

A general decline in mitochondrial function occurs as organisms age (reviewed in Lopez‐Otin, Blasco, Partridge, Serrano & Kroemer, 2013). In a paradoxical way, in yeast (Delaney et al., 2013), worms (Dillin et al., 2002), flies (Owusu‐Ansah, Song & Perrimon, 2013), and mice (Liu et al., 2005), suppression of mitochondrial ETC function increases lifespan (Dell'agnello et al., 2007; Durieux, Wolff & Dillin, 2011). While counterintuitive, these findings are supported by reports demonstrating that upregulation of mitochondrial stress response contributes to enhanced longevity in the long‐lived mitochondrial mutants (Durieux et al., 2011; Kirchman, Kim, Lai & Jazwinski, 1999). A link between mtUPR and longevity was first revealed in two long‐lived *C. elegans* mitochondrial ETC mutants (*isp‐1* and *clk‐1*; Durieux et al., 2011). RNAi knockdown of either UBL5 or DVE1 (mediators of mtUPR) reversed lifespan extension in both mutants. Similar to that, increased longevity by muscle‐specific disruption of ETC Complex I in Drosophila was dependent on mtUPR (Owusu‐Ansah et al., 2013), and *Surf1* knockout mice deficient for ETC Complex IV had increased expression of mtUPR genes (Dell'agnello et al., 2007; Pulliam et al., 2014). It is important that, a number of other pro‐longevity models, such as NAD+/Sirtuin1 or rapamycin in *C. elegans*, also require mtUPR (Houtkooper et al., 2013; Owusu‐Ansah et al., 2013). It is likely that CLPP deficiency in oocytes results in a compromised mitochondrial stress response contributing to accumulation of damaged proteins, reduced oxidative phosphorylation, increased reactive oxidative stress production, and culminates in oocyte dysfunction and accelerated follicular depletion.

After characterizing the developmental and metabolic changes that occur in CLPP‐deficient oocytes and histomorphometric findings consistent with accelerated ovarian follicular depletion, we adopted an unbiased approach to identify genes and pathways affected by CLPP and performed RNAseq analysis in 3 and 6 months *Clpp* ^−/−^ GV stage oocytes, compared to WT (Figure 5). We found 124 genes to be differentially expressed at 3 months and 239 at 6 months, with 64 (20.4%) conserved between the two time points (Figure 5l). Pathways of cell death and survival, cellular and embryonic development, protein synthesis, and mitochondrial function were consistently affected (Figure 5d,h). In addition, RNAseq revealed altered expression of cell senescence‐related genes at 3 (*Gpx6*,* Lamtor1* and *Hist1h1e*) and 6 months (*Ctnnb1*,* Dyrk1a*,* Id3*,* Kdm2*,* Sin3b*) in *Clpp* ^−/−^ oocytes. Most importantly, we detected altered expression of genes regulating mTOR signaling (Figure 5k).

Mechanistic target of rapamycin is a serine/threonine protein kinase of the phosphatidylinositol‐3‐OH‐kinase (PI(3)K)‐related family that functions as a master regulator of cellular growth and metabolism in response to nutrient and hormonal cues (reviewed in Johnson, Rabinovitch & Kaeberlein, 2013). Mechanistic target of rapamycin functions in two different complexes: mTORC1 and mTORC2. Rapamycin, which inhibits the mTORC1, significantly extends lifespan in a number of model systems including mice (Harrison et al., 2009). In addition, mTORC1 inhibits autophagy in both yeast and mammalian cells (reviewed in Wei, Zhang, Cai & Xu, 2015), while both mTORC1 and mTORC2 regulate growth and proliferation (reviewed in Johnson et al., 2013; Saxton & Sabatini, 2017). We therefore assessed pS6 (downstream mediator of mTORC1) and pAKT473 (downstream mediator of mTORC2) activity at 3‐ and 6‐month‐old *Clpp* ^−/−^ mice ovaries and oocytes compared to WT (Figure 6). We observed a significant upregulation of pS6 at 6 months. pAKT 473 was also increased at 6 months, but to a lesser extent. We further assessed pS6K, p4EBP1 (downstream mediators of mTORC1), and p‐mTOR2481 (downstream mediator of mTORC2) activity at 6‐month‐old *Clpp* ^−/−^ mice ovaries; they were similarly upregulated. Then, we performed rescue experiments with rapamycin, an mTOR inhibitor. Rapamycin treatment has been reported to prolong ovarian lifespan (Dou et al., 2017), and inhibition of mTORC1 or mTORC1/2 within ovaries during chemotherapy co‐treatment resulted in preservation of primordial follicle counts (Goldman et al., 2017). In the current study, oocyte competence was significantly improved both in vivo and in vitro by rapamycin treatment (Figure 7). These findings collectively suggest that increased mTOR activation in *Clpp* ^−/−^ mice is at least partially responsible for their reproductive phenotype. It is also possible for rapamycin treatment to rescue the increased follicular atresia and accelerated follicular depletion phenotype as has recently been suggested for the *Fmr1* knockout mice (Mok‐Lin et al., 2018). These interactions as well as the impact of rapamycin treatment on fertilization, pre‐implantation embryo development, and fertility remain to be investigated.

In this study, we have two important and potentially related findings regarding CLPP's role on female reproduction. First, we find that *Clpp*–deficiency results in female infertility due to impaired oocyte and early embryo development. Second, we observe that targeted deletion of *Clpp* results in accelerated follicular depletion, which could represent a phenotype reminiscent of premature ovarian aging in *Clpp* ^−/−^ mice, especially within the context of mTOR activation and chromosome misalignment on oocyte spindles. Individual contributions of granulosa/cumulus and oocyte dysfunction to infertility and follicular depletion and the molecular mechanisms leading to observed changes in gene expression remain to be studied using cell‐specific knockout models.

## 4 EXPERIMENTAL PROCEDURES

An expanded section describing experimental procedures is available in Supporting Information Data S1.

### 4.1 Statistical analysis

Data are representative of at least three independent experiments unless otherwise specified. All statistical analyses were performed using Graph Pad Prism software and significance was assessed at *p *<* *0.05. Values were analyzed either by Student's *t* test, One‐way ANOVA, or Two‐way ANOVA as described in each figure legend.

## CONFLICT OF INTEREST

None declared.

## AUTHORS’ CONTRIBUTION

TW and ES designed this study and wrote the manuscript. TW, EB, ZJ, GL, MZ, and EE performed the experiments. TH and ES supervised the study.
