# Supplementary figures and images for: Mitochondrial unfolded protein response gene Clpp is required to maintain ovarian follicular reserve during aging, for oocyte competence, and development of pre‐implantation embryos
Source: Aging Cell. 2018 May 30;17(4):e12784. doi: 10.1111/acel.12784 (PMC6052477; doi:10.1111/acel.12784)

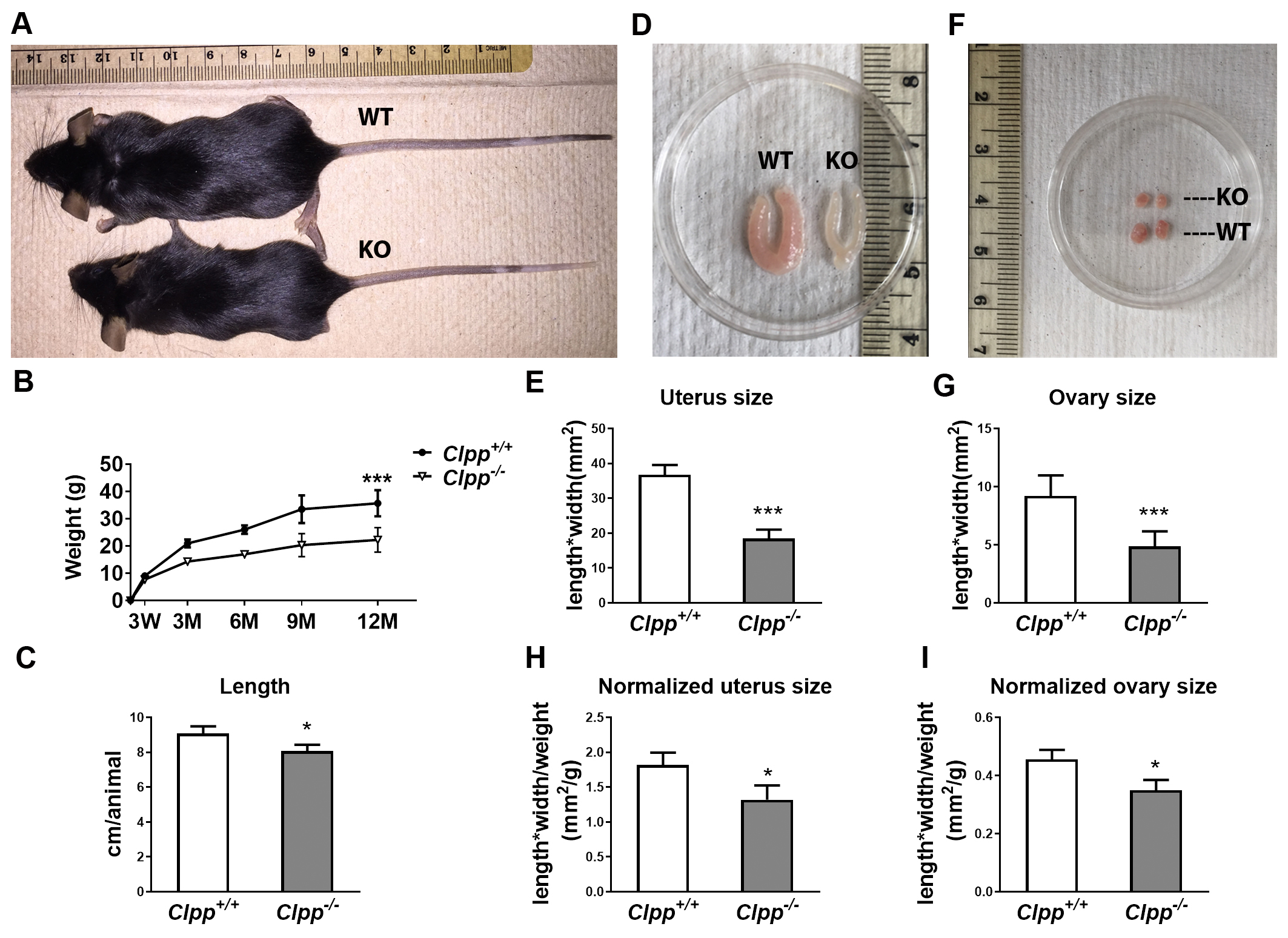

Supplement: Supplementary file 1 [file ACEL-17-na-s001.jpg]

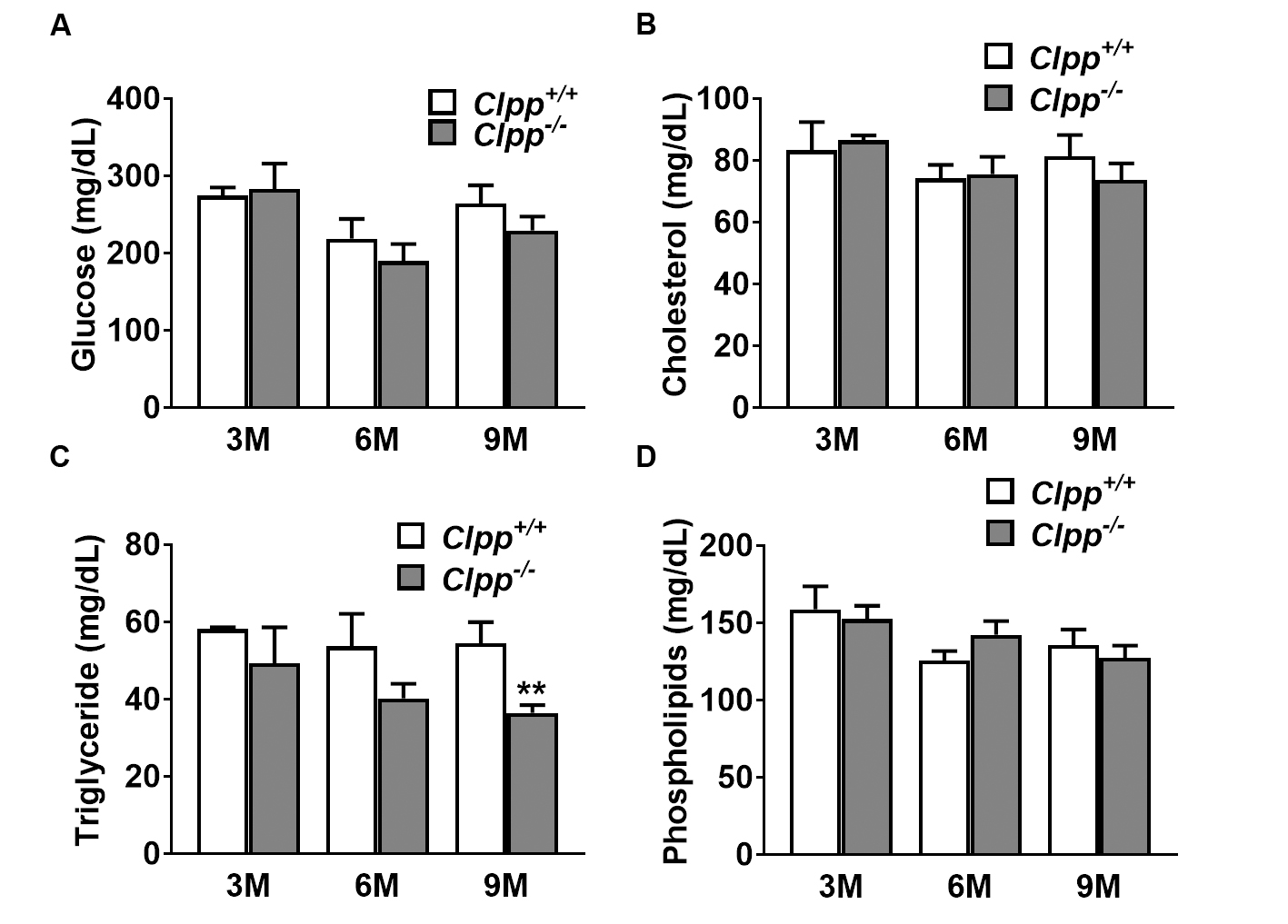

Supplement: Supplementary file 2 [file ACEL-17-na-s002.jpg]

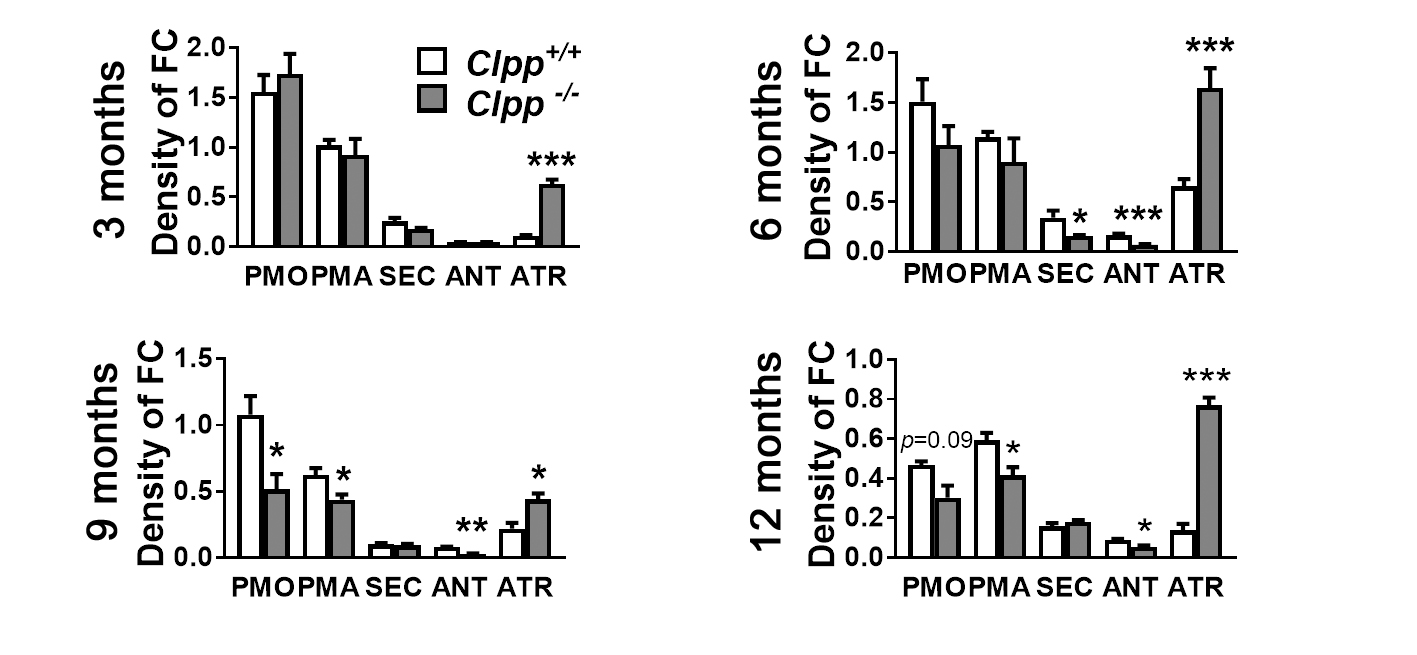

Supplement: Supplementary file 3 [file ACEL-17-na-s003.jpg]

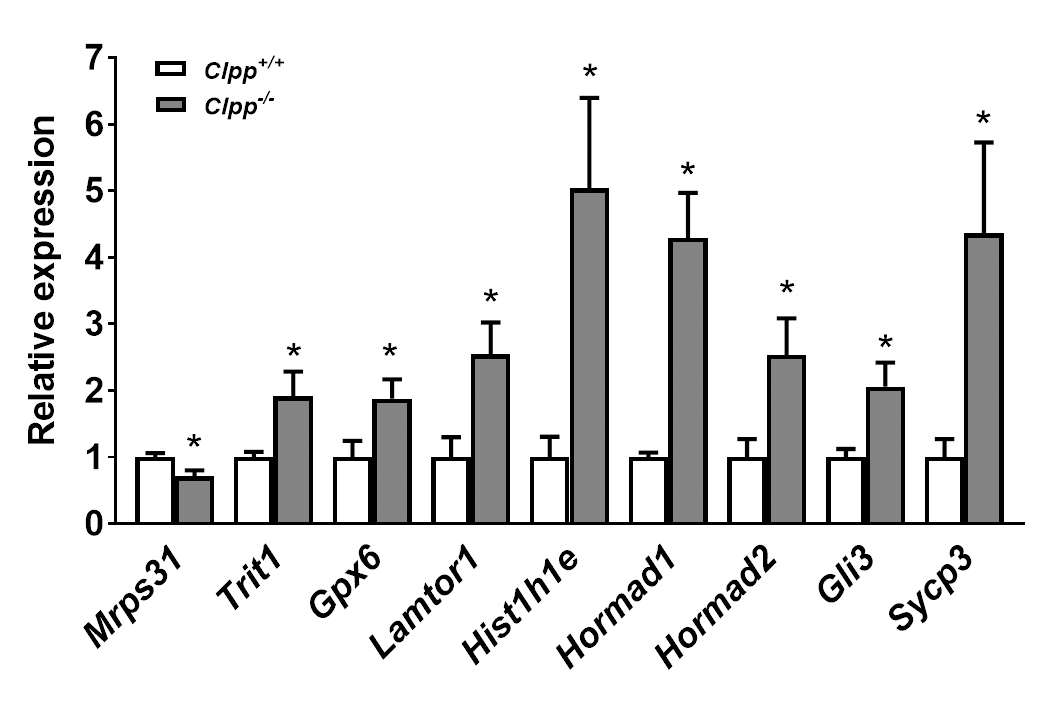

Supplement: Supplementary file 4 [file ACEL-17-na-s004.jpg]

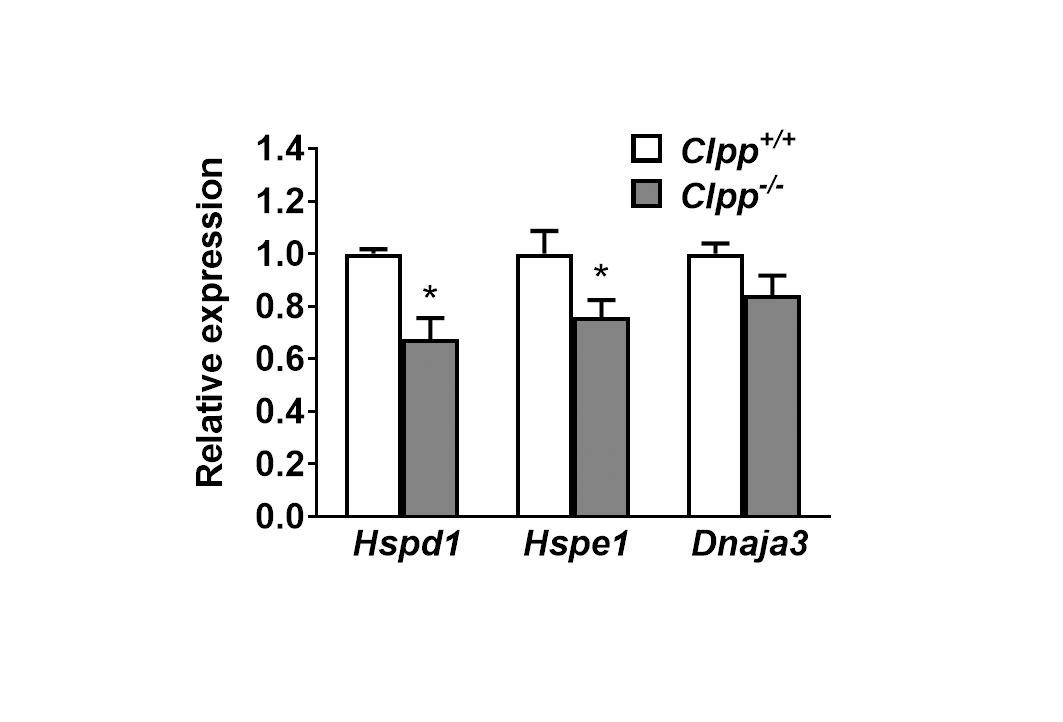

Supplement: Supplementary file 5 [file ACEL-17-na-s005.jpg]

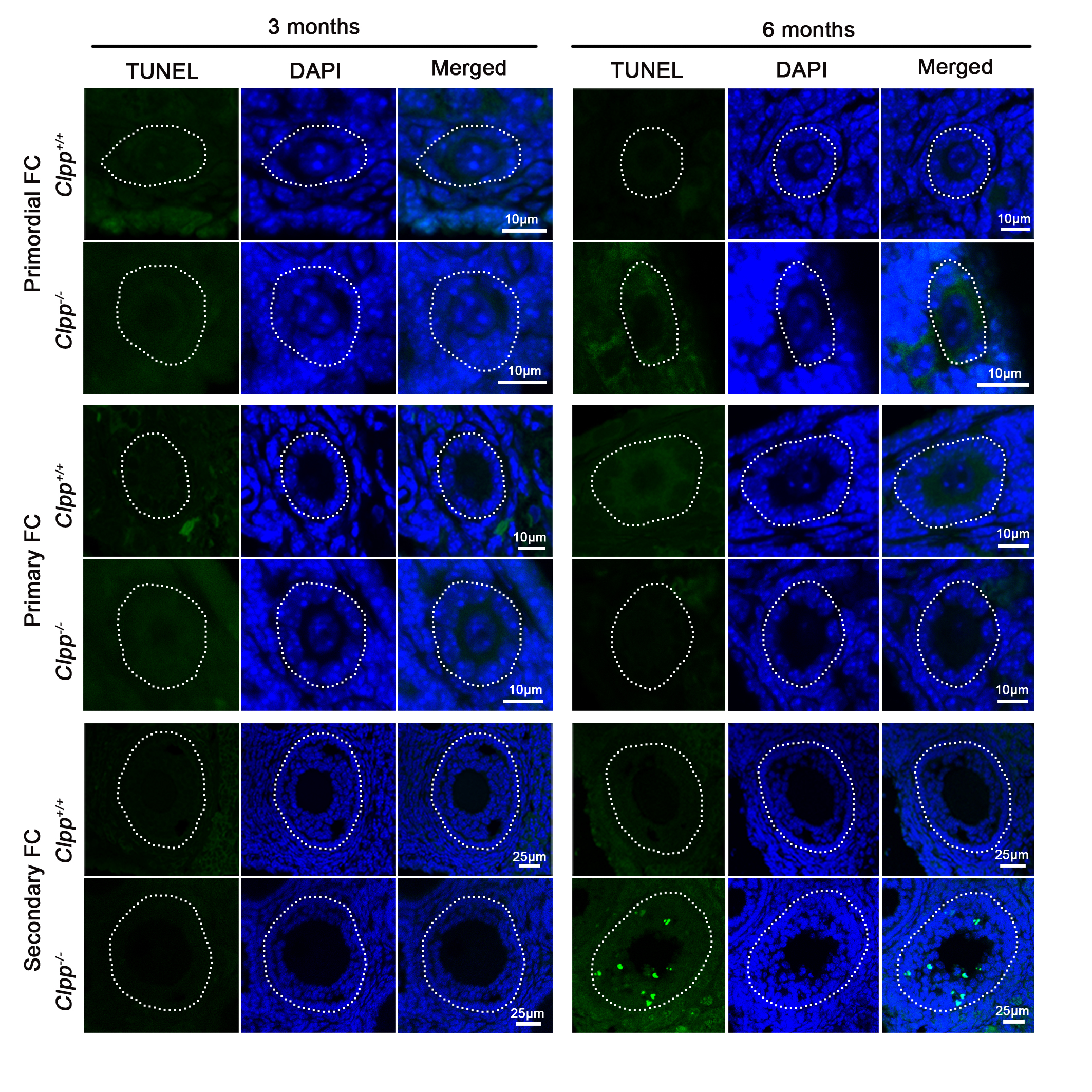

Supplement: Supplementary file 6 [file ACEL-17-na-s006.jpg]

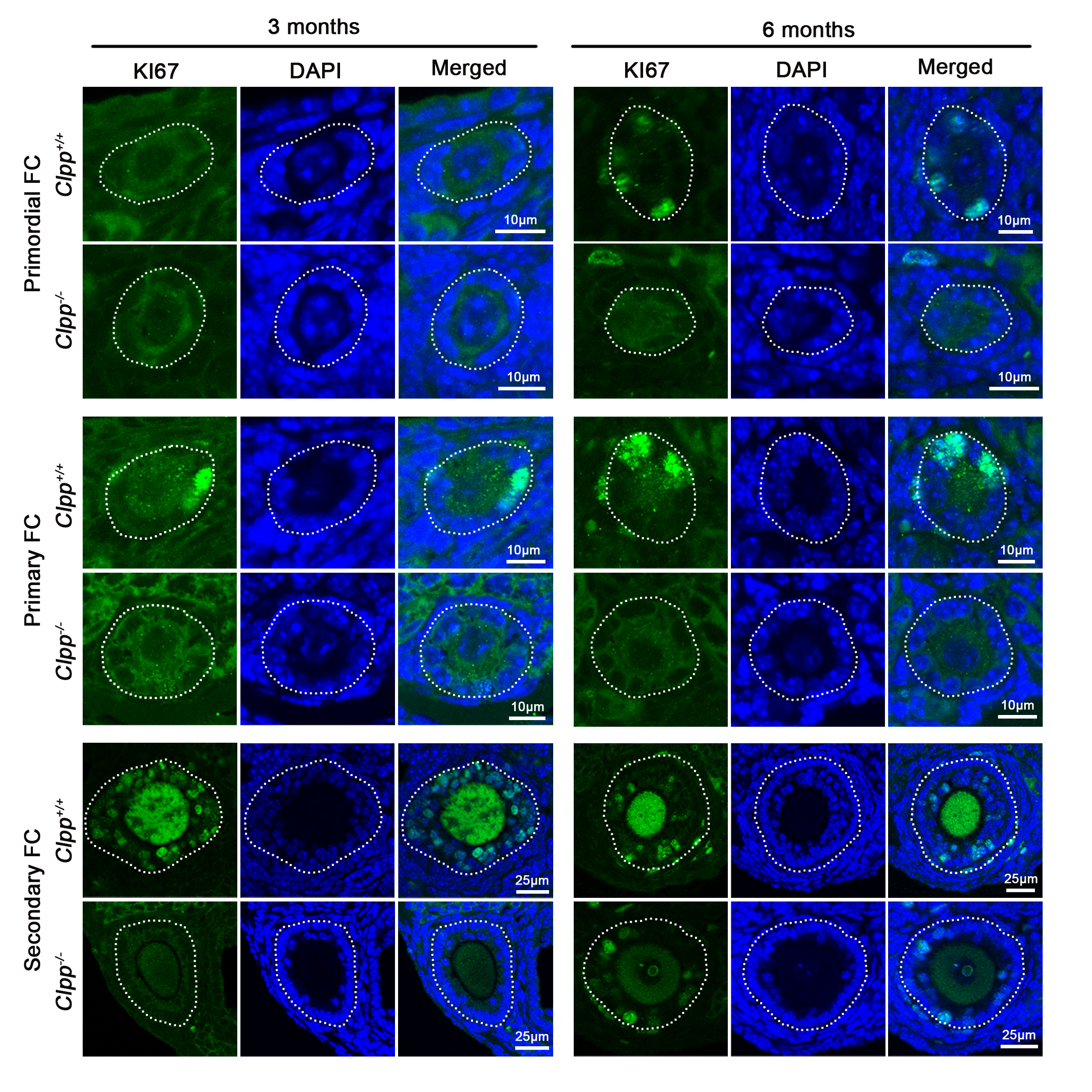

Supplement: Supplementary file 7 [file ACEL-17-na-s007.jpg]
