# Supplementary material for: Mitochondrial unfolded protein response gene Clpp is required to maintain ovarian follicular reserve during aging, for oocyte competence, and development of pre‐implantation embryos
Source: Aging Cell. 2018 May 30;17(4):e12784. doi: 10.1111/acel.12784 (PMC6052477; doi:10.1111/acel.12784)
Supplement: Supplementary file 8 [file ACEL-17-na-s008.docx]

**Table S1. The list of primers used for quantitative RT-PCR.**

| **Gene** | **TaqMan assay number or Primer sequences**  **(5' to 3'; F, forward; R, reverse)** |
| --- | --- |
| *β-actin* | Mm00607939_s1 |
| *β-actin* | F: GGCTGTATTCCCCTCCATCGR: CCAGTTGGTAACAATGCCATGT |
| *Atp5a1* | Mm00431960_m1 |
| *Bmp15* | Mm00437797_m1 |
| *Cox1* | Mm04225243_g1 |
| *Cox3* | F: TTTGCAGGATTCTTCTGAGCR: TGAGCTCATGTAATTGAAACACC |
| *Drp1* | F: TCCCAATTCCATTATCCTCGCR: CATCAGTACCCGCATCCATG |
| *Gapdh* | Mm99999915_ g1 |
| *Gdf9* | Mm00433565_m1 |
| *Gli3* | F: CACAGCTCTACGGCGACTGR: CTGCATAGTGATTGCGTTTCTTC |
| *Gpx6* | F: GCCCAGAAGTTGTGGGGTTCR: TCCATACTCATAGACGGTGCC |
| *Hist1h1e* | F: AGGCAAAGGCAACTAAGGCTAR: CTTTAGGCTTTACCGTTTTCGC |
| *Hormad1* | F: GGCTCCTAGCTGTTTCAGTATCTR: TTGTCCCATAAGCACGTTCTG |
| *Hormad2* | F: CCGGGAAGACAAAAAGTGTCCR: TGCTGCTGTCAAAGTCCATAG |
| *Lamtor1* | F: AGCAGCGAAAACGAGGACTCR: TGAAGGTAGGCTATGGTAGTTGG |
| *Mfn1* | Mm00612599_m1 |
| *Mfn2* | Mm00500120_m1 |
| *MRPS31* | F: CTCCACAGAATCCCGGCATTTR: ACTGGTCAACTTTCTTGCTACAG |
| *Ndufv1* | Mm00504941_m1 |
| *Opa1* | Mm01349707_g1 |
| *Sdhb* | Mm00458272_m1 |
| *Sycp3* | F: AGCCAGTAACCAGAAAATTGAGCR: CCACTGCTGCAACACATTCATA |
| *Trit1* | F: TCGGAAAGTAGAGCTTGAAAAGGR: ACCTGGCTTAGCCGTTTATGG |
| *Uqcrc2* | Mm00445961_m1 |

Abbreviations: *Atp5a1*: ATP synthase, H+ transporting, mitochondrial F1 complex, alpha subunit 1; *Bmp15*: bone morphogenetic protein 15; *Cox1*: cytochrome c oxidase subunit I; *Cox3*: cytochrome c oxidase subunit III; *Drp1:* Dynamin related protein 1;*Gapdh*, glyceraldehyde-3-phosphate dehydrogenase; *Gdf9*: growth differentiation factor 9; *Gli3*: GLI-Kruppel family member GLI3; *Gpx6*: glutathione peroxidase 6; *Hist1h1e*: histone cluster 1, H1e; *Hormad1*: HORMA domain containing 1; *Hormad2*: HORMA domain containing 2; *Lamtor1*: late endosomal/lysosomal adaptor, MAPK and MTOR activator 1; *Mfn1*: mitofusin 1; *Mfn2*: mitofusin 1; *Mrps31*: mitochondrial ribosomal protein S31; Trit1: tRNA isopentenyltransferase 1; *Ndufv1*: NADH dehydrogenase (ubiquinone) flavoprotein 1; *Opa1*: mitochondrial dynamin like GTPase; *Sdhb*: succinate dehydrogenase complex iron sulfur subunit B; *Sycp3*: synaptonemal complex protein 3; *Uqcrc2*: ubiquinol cytochrome c reductase core protein 2.
