# Supplementary material for: Mitochondrial unfolded protein response gene Clpp is required to maintain ovarian follicular reserve during aging, for oocyte competence, and development of pre‐implantation embryos
Source: Aging Cell. 2018 May 30;17(4):e12784. doi: 10.1111/acel.12784 (PMC6052477; doi:10.1111/acel.12784)
Supplement: Supplementary file 9 [file ACEL-17-na-s009.docx]

**Table S2 Significant deferentially expressed genes in 3 months GV (*ClpP*^-/-^ VS *ClpP*^+/+^)**

|  | Gene name | 3M_GV_KO | 3M_GV_WT | Foldchange |
| --- | --- | --- | --- | --- |
| Up-regulated genes | *Ptgds* | 89.2326 | 8.62762 | 10.34266692 |
|  | *Hormad1* | 6.6428 | 0.734279 | 9.046697509 |
|  | *Slc24a4* | 2.93653 | 0.391531 | 7.500121319 |
|  | *Gnpda1* | 2.71511 | 0.362198 | 7.496203734 |
|  | *Hist1h1e* | 15.9663 | 2.87154 | 5.560187217 |
|  | *Cdh8* | 3.60233 | 0.651156 | 5.532207336 |
|  | *Camk1g* | 1.76308 | 0.364683 | 4.834554942 |
|  | *Slc16a4* | 1.9796 | 0.440958 | 4.489316443 |
|  | *Serpinb1a* | 3.58017 | 0.816415 | 4.385233 |
|  | *Pls3* | 7.68366 | 1.90912 | 4.024712957 |
|  | *Hmgn3* | 19.0678 | 4.84483 | 3.93570053 |
|  | *Fam174b* | 2.15889 | 0.582484 | 3.706350732 |
|  | *Ceacam2* | 21.1102 | 5.95885 | 3.542663433 |
|  | *Sycp3* | 143.095 | 41.6055 | 3.439328935 |
|  | *Gpr143* | 11.0142 | 3.27258 | 3.365601452 |
|  | *Usp13* | 4.48447 | 1.35658 | 3.305717319 |
|  | *Wls* | 17.1512 | 5.34362 | 3.209659369 |
|  | *Arih2* | 3.02399 | 0.948273 | 3.188944534 |
|  | *Pepd* | 4.50303 | 1.43247 | 3.143542273 |
|  | *Zscan4d* | 27.1245 | 8.65376 | 3.134417872 |
|  | *Gm4907* | 56.2906 | 18.8165 | 2.991555284 |
|  | *Fxyd6* | 18.8106 | 6.40167 | 2.938389514 |
|  | *Ftl1* | 705.594 | 244.78 | 2.882563935 |
|  | *Gli3* | 6.53717 | 2.3226 | 2.814591406 |
|  | *Hormad2* | 10.5778 | 3.79802 | 2.785082754 |
|  | *Gstp2* | 39.5044 | 14.6216 | 2.701783663 |
|  | *Xlr3c* | 50.6129 | 18.8237 | 2.688785945 |
|  | *Tmod4* | 23.6403 | 8.94409 | 2.643119647 |
|  | *Ust* | 8.33616 | 3.34441 | 2.492565206 |
|  | *Gdpd3* | 14.5126 | 6.04181 | 2.402028531 |
|  | *Tmem159* | 18.9102 | 8.1915 | 2.308514924 |
|  | *A530046M15Rik* | 20.2102 | 9.22047 | 2.191883928 |
|  | *Ceacam10* | 57.5624 | 26.2747 | 2.190791902 |
|  | *Serpini1* | 118.824 | 55.1561 | 2.154322006 |
|  | *Steap4* | 11.0384 | 5.19341 | 2.125462846 |
|  | *Nif3l1* | 44.643 | 21.126 | 2.113178074 |
|  | *Ablim3* | 4.74378 | 2.24797 | 2.110250582 |
|  | *Faxc* | 4.42279 | 2.09711 | 2.108992852 |
|  | *Fkbp6* | 64.1674 | 30.5378 | 2.101245014 |
|  | *Gpx6* | 158.107 | 76.3143 | 2.071787332 |
|  | *Tmem184c* | 32.6296 | 15.9941 | 2.040102288 |
|  | *Nudt9* | 95.6644 | 47.3482 | 2.020444283 |
|  | *Cep128* | 8.87427 | 4.41407 | 2.010450673 |
|  | *Gstp1* | 155.563 | 77.7725 | 2.000231444 |
|  | *Lap3* | 12.7049 | 6.49991 | 1.954627064 |
|  | *Psg16* | 29.1498 | 15.1195 | 1.927960581 |
|  | *Ell3* | 46.2555 | 24.4688 | 1.890386942 |
|  | *Ell2* | 9.85033 | 5.26778 | 1.869920536 |
|  | *Eya1* | 21.1786 | 11.3716 | 1.862411622 |
|  | *Cyp39a1* | 26.3674 | 14.253 | 1.849954396 |
|  | *Sat1* | 112.782 | 61.1342 | 1.844826627 |
|  | *Serinc1* | 66.7321 | 36.6174 | 1.822415027 |
|  | *Chmp2b* | 48.4552 | 26.7386 | 1.81218164 |
|  | *Ttc39b* | 4.14523 | 2.29146 | 1.808990774 |
|  | *Mef2b* | 55.1969 | 30.5975 | 1.803967644 |
|  | *Atp6ap2* | 56.0728 | 31.2803 | 1.792591503 |
|  | *Csmd3* | 8.33034 | 4.64823 | 1.792153142 |
|  | *Stam2* | 12.6867 | 7.10002 | 1.786854122 |
|  | *Tubal3* | 58.6324 | 32.9824 | 1.777687494 |
|  | *Hmgb3* | 104.417 | 58.9742 | 1.770553903 |
|  | *Stard4* | 16.9937 | 9.61204 | 1.767959767 |
|  | *Cldn15* | 37.8688 | 21.6236 | 1.751271759 |
|  | *Il10rb* | 41.8642 | 23.9203 | 1.750153635 |
|  | *Slc10a6* | 45.7133 | 26.1322 | 1.749309281 |
|  | *Hat1* | 309.376 | 181.566 | 1.703931353 |
|  | *Naip1* | 20.3381 | 12.0252 | 1.691289958 |
|  | *Taf9b* | 267.732 | 159.306 | 1.680614666 |
|  | *Lamtor1* | 205.39 | 123.523 | 1.662767258 |
|  | *Trit1* | 55.3185 | 33.2744 | 1.66249429 |
|  | *Tfg* | 47.6961 | 28.7427 | 1.65941613 |
|  | *Tank* | 102.84 | 62.1672 | 1.654248543 |
|  | *Ccnb2* | 284.678 | 172.838 | 1.647079925 |
|  | *Prex2* | 23.8342 | 14.6757 | 1.624058818 |
| Down-regulated genes | *Tet3* | 15.6216 | 25.3456 | 0.616343665 |
|  | *Kbtbd7* | 29.2803 | 47.5585 | 0.615669123 |
|  | *Slc7a14* | 10.7793 | 17.5938 | 0.612676056 |
|  | *Mrps31* | 101.532 | 166.248 | 0.610726144 |
|  | *Luc7l2* | 24.0656 | 39.5231 | 0.608899606 |
|  | *Ctnnb1* | 34.3391 | 56.8115 | 0.604439242 |
|  | *Arid1a* | 9.1188 | 15.1834 | 0.600576946 |
|  | *Mtss1* | 9.41607 | 15.7043 | 0.599585464 |
|  | *Casc4* | 22.2449 | 37.4478 | 0.594024215 |
|  | *Kdm2b* | 28.8342 | 48.6712 | 0.592428377 |
|  | *Nmrk1* | 38.0763 | 64.5933 | 0.589477546 |
|  | *Hnrnpul2* | 10.4698 | 18.063 | 0.579626862 |
|  | *Tor4a* | 21.3783 | 36.953 | 0.578526777 |
|  | *Chsy1* | 15.8252 | 27.3688 | 0.578220455 |
|  | *Rab33b* | 9.95378 | 17.3455 | 0.573853737 |
|  | *Slain1* | 17.8852 | 31.4566 | 0.568567487 |
|  | *Myh11* | 6.48024 | 11.6002 | 0.558631748 |
|  | *Khdc1a* | 20.8643 | 37.8398 | 0.55138505 |
|  | *Ubqln2* | 5.96856 | 10.9971 | 0.542739449 |
|  | *Heatr2* | 5.77139 | 10.8459 | 0.532126426 |
|  | *Cstb* | 51.0602 | 96.7502 | 0.527752914 |
|  | *Fmnl3* | 17.3349 | 33.129 | 0.52325455 |
|  | *Zbtbd6* | 5.83269 | 11.194 | 0.521055029 |
|  | *Itgb3* | 3.09333 | 5.94949 | 0.519931961 |
|  | *Aph1c* | 4.76372 | 9.33615 | 0.510244587 |
|  | *Rapgef3* | 7.83588 | 15.4462 | 0.507301472 |
|  | *Cd200* | 8.71936 | 17.2413 | 0.505725206 |
|  | *Ly6e* | 21.7535 | 43.6696 | 0.498138293 |
|  | *Taf4a* | 3.49726 | 7.04479 | 0.496432115 |
|  | *Tac2* | 105.279 | 215.888 | 0.487655636 |
|  | *Krt12* | 37.5618 | 77.2101 | 0.486488167 |
|  | *Brd1* | 6.76236 | 14.394 | 0.469804085 |
|  | *Ddx58* | 4.45482 | 9.69229 | 0.459625125 |
|  | *Gm6083* | 22.4511 | 49.0433 | 0.457781185 |
|  | *Sipa1l2* | 1.40576 | 3.0904 | 0.454879627 |
|  | *2010107G23Rik* | 25.4687 | 56.5906 | 0.450051775 |
|  | *Fam84a* | 2.39193 | 5.34719 | 0.44732467 |
|  | *Gm5531* | 5.62564 | 13.1064 | 0.429228469 |
|  | *Dcpp3* | 29.51 | 70.8608 | 0.41645028 |
|  | *Nav1* | 0.820962 | 1.98247 | 0.41411068 |
|  | *Arhgef28* | 1.44438 | 3.51138 | 0.411342549 |
|  | *Cxcr3* | 11.4183 | 30.9601 | 0.368806948 |
|  | *Stard5* | 4.32298 | 11.7753 | 0.367122706 |
|  | *Crabp2* | 186.862 | 512.161 | 0.364850115 |
|  | *Glis1* | 4.20718 | 12.2378 | 0.343785648 |
|  | *Gm14164* | 2.21891 | 6.66376 | 0.33298168 |
|  | *Ptpn3* | 0.478324 | 1.50996 | 0.316779252 |
|  | *Plek* | 1.30393 | 4.20061 | 0.31041444 |
|  | *Krt73* | 2.08767 | 7.51111 | 0.277944272 |
|  | *Dirc2* | 1.06391 | 3.91117 | 0.272018347 |
|  | *Clpp* | 0.963185 | 26.0125 | 0.037027775 |
