# Supplementary material for: Mitochondrial unfolded protein response gene Clpp is required to maintain ovarian follicular reserve during aging, for oocyte competence, and development of pre‐implantation embryos
Source: Aging Cell. 2018 May 30;17(4):e12784. doi: 10.1111/acel.12784 (PMC6052477; doi:10.1111/acel.12784)
Supplement: Supplementary file 10 [file ACEL-17-na-s010.docx]

**Table S3 Significant deferentially expressed genes in 6 months GV (*ClpP*^-/-^ VS *ClpP*^+/+^)**

|  | **Gene name** | **6M_GV_KO** | **6M_GV_WT** | **Foldchange** |
| --- | --- | --- | --- | --- |
| Up-regulated genes | *Herpud1* | 9.66993 | 0.181527 | 53.26992679 |
|  | *Psat1* | 2.40741 | 0.183332 | 13.13142277 |
|  | *Ptgds* | 55.7525 | 5.47294 | 10.18693792 |
|  | *Mterfd3* | 5.47086 | 0.694803 | 7.87397291 |
|  | *Hormad1* | 5.80129 | 0.852929 | 6.801609513 |
|  | *Zfp772* | 2.00488 | 0.31517 | 6.361265349 |
|  | *Gnpda1* | 2.63266 | 0.420712 | 6.257629923 |
|  | *Slc24a4* | 3.13636 | 0.552139 | 5.680381208 |
|  | *Hist1h1e* | 15.7905 | 3.22546 | 4.895580785 |
|  | *Pepd* | 3.99126 | 0.822418 | 4.853079578 |
|  | *Vimp* | 6.88486 | 1.46738 | 4.691940738 |
|  | *Hmgn3* | 13.6962 | 2.94613 | 4.648878359 |
|  | *Chchd4* | 5.16188 | 1.13159 | 4.56161684 |
|  | *Sord* | 1.9313 | 0.427358 | 4.51916192 |
|  | *Hmox1* | 2.83859 | 0.647603 | 4.383225526 |
|  | *Etfdh* | 3.31301 | 0.768357 | 4.311810786 |
|  | *Pls3* | 9.16622 | 2.13203 | 4.299292224 |
|  | *Alg5* | 2.93458 | 0.684233 | 4.288860666 |
|  | *Atp4a* | 3.42722 | 0.831283 | 4.122807756 |
|  | *Dpf2* | 4.62504 | 1.13956 | 4.058619116 |
|  | *Wls* | 15.803 | 4.01043 | 3.940475211 |
|  | *Jade3* | 1.76824 | 0.499161 | 3.542424188 |
|  | *Ldhc* | 34.7416 | 10.1092 | 3.436631979 |
|  | *Gstm1* | 12.6212 | 3.69095 | 3.419499045 |
|  | *Arl2bp* | 5.9027 | 1.74171 | 3.389025728 |
|  | *Psmg2* | 8.65739 | 2.61207 | 3.314379017 |
|  | *Cdh8* | 3.25062 | 0.981263 | 3.31268987 |
|  | *Npdc1* | 5.00851 | 1.56559 | 3.199119821 |
|  | *Eif4ebp1* | 17.6889 | 5.59669 | 3.160600283 |
|  | *Tex19.2* | 5.6227 | 1.7935 | 3.135043212 |
|  | *Asns* | 6.61047 | 2.11573 | 3.124439319 |
|  | *Gstm2* | 17.9518 | 5.85176 | 3.06776081 |
|  | *Ceacam2* | 19.0755 | 6.27626 | 3.039310035 |
|  | *Zfp263* | 2.9281 | 0.963901 | 3.037760102 |
|  | *Id3* | 40.8374 | 13.6541 | 2.990852564 |
|  | *Zfp707* | 10.0602 | 3.38708 | 2.970168995 |
|  | *Spcs3* | 6.34826 | 2.18996 | 2.898801805 |
|  | *Ftl1* | 606.709 | 211.252 | 2.871968076 |
|  | *Ubtf* | 4.97071 | 1.73426 | 2.866185001 |
|  | *Nxt2* | 6.48709 | 2.32265 | 2.792969238 |
|  | *Eif1a* | 4.25452 | 1.53033 | 2.78013239 |
|  | *Hormad2* | 8.74411 | 3.21865 | 2.716701101 |
|  | *Clp1* | 7.05195 | 2.60816 | 2.703802681 |
|  | *0610031J06Rik* | 10.8215 | 4.02451 | 2.688898773 |
|  | *Yrdc* | 7.97167 | 2.97863 | 2.676287421 |
|  | *G2e3* | 1.99798 | 0.746706 | 2.675725118 |
|  | *Tuft1* | 4.78638 | 1.78895 | 2.675524749 |
|  | *Zfx* | 3.01447 | 1.13431 | 2.6575363 |
|  | *Uqcrc1* | 18.8954 | 7.24462 | 2.608197531 |
|  | *Cct6a* | 15.0716 | 5.81851 | 2.590285142 |
|  | *Mcfd2* | 18.8621 | 7.33526 | 2.571428961 |
|  | *Pnrc2* | 7.83186 | 3.06239 | 2.5574339 |
|  | *Nlrp14* | 4154.47 | 1625.73 | 2.555448937 |
|  | *Zp2* | 2993.92 | 1195.07 | 2.505225635 |
|  | *Atp6v0e2* | 11.0796 | 4.44497 | 2.492615248 |
|  | *Slc30a6* | 9.20774 | 3.71626 | 2.477689936 |
|  | *Ablim3* | 5.5503 | 2.36028 | 2.351543037 |
|  | *4930470H14Rik* | 15.4294 | 6.62395 | 2.329335215 |
|  | *Ctsd* | 19.674 | 8.52532 | 2.307713963 |
|  | *Ceacam10* | 40.1761 | 17.5071 | 2.294846091 |
|  | *C1d* | 7.25826 | 3.16335 | 2.294485277 |
|  | *Nlrp5* | 616.729 | 270.197 | 2.28251609 |
|  | *Padi6* | 2162.3 | 954.108 | 2.266305282 |
|  | *Haus5* | 13.2906 | 5.87213 | 2.263335451 |
|  | *Sycp3* | 83.265 | 37.07 | 2.246155921 |
|  | *Rab11b* | 6.72643 | 2.99501 | 2.245878979 |
|  | *Arf4* | 16.8876 | 7.64694 | 2.208412777 |
|  | *Wdr61* | 42.8493 | 19.5918 | 2.187103788 |
|  | *Tfrc* | 5.33444 | 2.44056 | 2.185744255 |
|  | *Tspan3* | 65.6458 | 30.0714 | 2.182997799 |
|  | *Pnldc1* | 33.0459 | 15.2499 | 2.166958472 |
|  | *Atp6ap2* | 47.9755 | 22.2638 | 2.154865746 |
|  | *Zfp473* | 22.6665 | 10.5321 | 2.152134902 |
|  | *AW209491* | 15.5691 | 7.2847 | 2.13723283 |
|  | *Stxbp3a* | 23.4031 | 11.0599 | 2.116031791 |
|  | *Ldhb* | 8613.84 | 4072.42 | 2.115164939 |
|  | *Fxyd6* | 16.6813 | 7.88679 | 2.11509372 |
|  | *Sin3b* | 180.278 | 85.5228 | 2.107952499 |
|  | *Serpini1* | 118.121 | 56.167 | 2.103032029 |
|  | *Wdr3* | 9.18744 | 4.37561 | 2.099693528 |
|  | *Ormdl1* | 77.566 | 36.9827 | 2.097359035 |
|  | *Cyp39a1* | 26.4118 | 12.6321 | 2.090847919 |
|  | *Ogt* | 31.9852 | 15.3203 | 2.087765905 |
|  | *Slc35b2* | 9.91182 | 4.75964 | 2.082472624 |
|  | *Tmem159* | 22.0745 | 10.6198 | 2.0786173 |
|  | *Rbm5* | 16.6257 | 8.0565 | 2.063638056 |
|  | *Ddx4* | 48.7793 | 23.6621 | 2.061494965 |
|  | *Xlr3c* | 38.8941 | 19.0492 | 2.041770783 |
|  | *Snrpb* | 36.4404 | 17.8516 | 2.041296018 |
|  | *Acadsb* | 19.3538 | 9.53055 | 2.030711764 |
|  | *Zfp62* | 16.1989 | 8.07301 | 2.006550221 |
|  | *Gm4907* | 45.3397 | 22.7983 | 1.988731616 |
|  | *Ell3* | 44.589 | 22.5003 | 1.981706911 |
|  | *Dynlt3* | 68.9812 | 34.8802 | 1.977660679 |
|  | *Aff4* | 2.9435 | 1.48936 | 1.976352259 |
|  | *B3galnt1* | 28.2151 | 14.4531 | 1.952183269 |
|  | *Paqr6* | 22.8746 | 11.7319 | 1.949777956 |
|  | *Ikbkb* | 20.4168 | 10.4928 | 1.9457914 |
|  | *Slc10a6* | 55.115 | 28.37 | 1.942721184 |
|  | *Gpx6* | 145.183 | 75.6391 | 1.919417338 |
|  | *Haus8* | 142.656 | 74.7941 | 1.907316219 |
|  | *Endod1* | 10.2926 | 5.41172 | 1.90190919 |
|  | *Slc25a39* | 21.5762 | 11.3858 | 1.895009573 |
|  | *Ddx6* | 12.6866 | 6.72168 | 1.887415051 |
|  | *Tmem184c* | 34.1432 | 18.1373 | 1.882485265 |
|  | *Nudt9* | 90.4714 | 48.1408 | 1.879308196 |
|  | *Il10rb* | 44.8843 | 23.8866 | 1.879057714 |
|  | *Hat1* | 265.442 | 141.984 | 1.869520509 |
|  | *Pnpla2* | 18.6248 | 10.035 | 1.855984056 |
|  | *Napa* | 56.3126 | 30.5063 | 1.845933463 |
|  | *Rrm1* | 21.275 | 11.7363 | 1.812751889 |
|  | *Vps41* | 44.8649 | 24.8162 | 1.80788759 |
|  | *Strada* | 61.1152 | 33.8145 | 1.807366662 |
|  | *Hmgb3* | 95.0438 | 52.6382 | 1.805605055 |
|  | *Ptpn6* | 25.1749 | 13.9654 | 1.802662294 |
|  | *Taf9b* | 297.925 | 165.877 | 1.796059731 |
|  | *Hpse* | 17.6781 | 9.87196 | 1.790738617 |
|  | *Zfp639* | 40.0847 | 22.4595 | 1.784754781 |
|  | *Tfg* | 53.3213 | 29.9011 | 1.783255466 |
|  | *Lamtor1* | 165.559 | 93.2793 | 1.774873954 |
|  | *Ccdc158* | 17.2458 | 9.74102 | 1.770430612 |
|  | *Dppa5a* | 2820.58 | 1595.28 | 1.768078331 |
|  | *Fbxl5* | 76.7843 | 43.4679 | 1.766459847 |
|  | *Coq7* | 87.1114 | 49.5936 | 1.756504872 |
|  | *Bzw1* | 45.3835 | 26.2076 | 1.731692334 |
|  | *Tcn2* | 31.4875 | 18.1916 | 1.730881286 |
|  | *Gstp1* | 135.558 | 78.5421 | 1.725927878 |
|  | *Pttg1ip* | 72.95 | 42.4287 | 1.719355059 |
|  | *Tubg2* | 81.383 | 47.3857 | 1.717459065 |
|  | *Tada1* | 69.4449 | 40.483 | 1.715408937 |
|  | *Cog6* | 29.7566 | 17.3478 | 1.715295311 |
|  | *Pecr* | 1301.41 | 761.026 | 1.71007298 |
|  | *Ccbl2* | 42.9326 | 25.1243 | 1.708807808 |
|  | *Serinc1* | 77.8854 | 45.7676 | 1.701758449 |
|  | *Morf4l2* | 209.582 | 124.133 | 1.68836651 |
|  | *Nif3l1* | 35.9784 | 21.324 | 1.687225661 |
|  | *Cse1l* | 35.4439 | 21.2078 | 1.671267175 |
|  | *Uqcrc2* | 312.661 | 187.919 | 1.663807279 |
|  | *Mfsd1* | 42.9784 | 25.8422 | 1.663109178 |
|  | *Rictor* | 30.7516 | 18.4989 | 1.662347491 |
|  | *Astl* | 99.667 | 60.0945 | 1.658504522 |
|  | *Enpp5* | 82.5667 | 49.8417 | 1.656578728 |
|  | *Actg1* | 137.678 | 83.1258 | 1.656260752 |
|  | *Tom1l1* | 60.8846 | 36.8171 | 1.653704393 |
|  | *Pdhb* | 92.6977 | 56.4644 | 1.641701674 |
|  | *Prkab1* | 89.1143 | 54.5223 | 1.634455993 |
|  | *Atp6v1b2* | 88.3993 | 54.2081 | 1.630739687 |
|  | *Ythdf2* | 34.4342 | 21.1831 | 1.625550557 |
|  | *Prr14* | 194.338 | 120.025 | 1.619146011 |
|  | *Cryl1* | 150.038 | 92.7993 | 1.616800989 |
|  | *Nup93* | 87.4719 | 54.2376 | 1.612753883 |
| Down-regulated genes | *Herc2* | 6.21696 | 10.0241 | 0.620201315 |
|  | *Ticrr* | 17.1716 | 27.8167 | 0.617312622 |
|  | *A230046K03Rik* | 18.7649 | 30.5554 | 0.614127126 |
|  | *Suz12* | 36.7867 | 59.9707 | 0.613411216 |
|  | *Sclt1* | 19.6559 | 32.1822 | 0.610769307 |
|  | *BC018507* | 26.5202 | 43.5591 | 0.608832598 |
|  | *Dyrk1a* | 14.3886 | 23.6911 | 0.607341998 |
|  | *Esyt2* | 13.9831 | 23.0247 | 0.607308673 |
|  | *Chsy1* | 21.0883 | 34.7784 | 0.606361995 |
|  | *Mapk8* | 12.7034 | 21.168 | 0.600122827 |
|  | *Krt12* | 41.43 | 69.3943 | 0.597023098 |
|  | *Gm17821* | 3.99155 | 6.69238 | 0.596432062 |
|  | *Gcnt4* | 7.83275 | 13.2143 | 0.592748008 |
|  | *Arid2* | 4.71365 | 7.96239 | 0.59198934 |
|  | *Itga6* | 24.6363 | 41.6688 | 0.591240928 |
|  | *Ctnnb1* | 30.5838 | 52.2142 | 0.585737213 |
|  | *Aim1* | 5.7112 | 9.76851 | 0.584654159 |
|  | *Gm16702* | 23.3516 | 39.9815 | 0.584060128 |
|  | *Cited2* | 39.4121 | 67.7474 | 0.581750739 |
|  | *Atp13a3* | 10.7823 | 18.6078 | 0.579450553 |
|  | *Arid1a* | 7.23132 | 12.4798 | 0.579441978 |
|  | *Kbtbd7* | 24.9922 | 43.242 | 0.577961241 |
|  | *Mrps31* | 96.9943 | 168.708 | 0.574924129 |
|  | *Aph1c* | 5.08528 | 8.86493 | 0.573640175 |
|  | *Intu* | 36.9153 | 64.3678 | 0.573505697 |
|  | *Sptssb* | 19.462 | 34.1756 | 0.569470616 |
|  | *Ppp1cb* | 7.75398 | 13.6978 | 0.566074844 |
|  | *Casc4* | 11.9159 | 21.1386 | 0.563703367 |
|  | *Tulp3* | 7.64925 | 13.6695 | 0.559585208 |
|  | *Khdc1a* | 19.2908 | 34.5951 | 0.557616541 |
|  | *Kdm6b* | 4.2891 | 7.69305 | 0.557529198 |
|  | *Ddx58* | 4.13424 | 7.41742 | 0.557369004 |
|  | *Fkbp5* | 15.0771 | 27.0757 | 0.556849869 |
|  | *Oca2* | 7.54327 | 13.5645 | 0.5561038 |
|  | *Alg10b* | 8.24077 | 14.8413 | 0.55525931 |
|  | *1810013L24Rik* | 5.68198 | 10.2352 | 0.555141082 |
|  | *Lrrc16a* | 5.53209 | 9.96861 | 0.554950991 |
|  | *Cnst* | 14.8017 | 26.6822 | 0.554740614 |
|  | *Atp8a2* | 12.891 | 23.7307 | 0.543220385 |
|  | *Mei4* | 13.7627 | 25.3602 | 0.542688938 |
|  | *Abl2* | 9.00655 | 16.6925 | 0.539556687 |
|  | *Heatr2* | 5.22654 | 9.71641 | 0.537908549 |
|  | *Loxl2* | 4.1994 | 7.93616 | 0.529147598 |
|  | *Prss12* | 19.1498 | 36.3382 | 0.526988128 |
|  | *Arf3* | 5.86512 | 11.1334 | 0.526804031 |
|  | *Slain1* | 14.9852 | 28.5918 | 0.524108311 |
|  | *Irak1* | 5.75239 | 11.047 | 0.520719652 |
|  | *Foxk2* | 3.83789 | 7.43683 | 0.516065313 |
|  | *Taf4a* | 3.49401 | 6.84519 | 0.510432873 |
|  | *Luc7l2* | 14.3614 | 28.1732 | 0.509753951 |
|  | *Hnrnpul2* | 8.75701 | 17.3179 | 0.505662349 |
|  | *Glis1* | 5.3079 | 10.5417 | 0.503514613 |
|  | *Slco1a5* | 13.1981 | 26.2829 | 0.502155394 |
|  | *Zbtbd6* | 5.01048 | 10.0208 | 0.500007983 |
|  | *Nkrf* | 3.96074 | 8.0028 | 0.494919278 |
|  | *Fat1* | 1.72167 | 3.48144 | 0.494528126 |
|  | *Kdm2b* | 22.755 | 46.1202 | 0.493384677 |
|  | *Zfp820* | 2.74146 | 5.65926 | 0.484420225 |
|  | *Tcf20* | 3.42775 | 7.08263 | 0.483965702 |
|  | *Rab33b* | 5.96988 | 12.3741 | 0.482449633 |
|  | *Cdh1* | 9.823 | 20.5848 | 0.477196767 |
|  | *Cpeb4* | 2.45193 | 5.18132 | 0.47322497 |
|  | *Cd200* | 7.06779 | 14.9627 | 0.472360603 |
|  | *Gm15645* | 17.7208 | 37.529 | 0.472189507 |
|  | *Rph3a* | 4.99056 | 10.7003 | 0.4663944 |
|  | *Csrnp3* | 0.733816 | 1.58476 | 0.463045508 |
|  | *Tmem69* | 5.33056 | 11.6135 | 0.458996857 |
|  | *Fam84b* | 2.74653 | 6.04362 | 0.45445114 |
|  | *Pthlh* | 5.97659 | 13.4708 | 0.443670012 |
|  | *Phf23* | 5.90276 | 13.3178 | 0.443223355 |
|  | *Ubqln2* | 5.15394 | 11.6824 | 0.441171335 |
|  | *Ung* | 4.52373 | 10.3386 | 0.43755731 |
|  | *Gatad2b* | 26.2623 | 60.8802 | 0.431376704 |
|  | *Dcpp3* | 20.9111 | 49.2714 | 0.424406451 |
|  | *2010107G23Rik* | 20.8373 | 49.4313 | 0.421540603 |
|  | *Cd5* | 7.82658 | 19.3196 | 0.405110872 |
|  | *Pard6g* | 2.6676 | 6.8596 | 0.388885649 |
|  | *Vmn2r-ps129* | 4.5019 | 12.3435 | 0.364718273 |
|  | *Gm5531* | 3.32778 | 9.66057 | 0.344470357 |
|  | *Slc44a3* | 2.10189 | 6.16281 | 0.341060328 |
|  | *Kbtbd11* | 0.636784 | 1.88621 | 0.337599737 |
|  | *Nfkbiz* | 1.84324 | 5.68564 | 0.324192175 |
|  | *Cdc5l* | 2.96892 | 9.18639 | 0.323186801 |
|  | *Plek* | 1.13074 | 3.5977 | 0.314295244 |
|  | *Crabp2* | 155.687 | 527.566 | 0.295104309 |
|  | *Plac1* | 3.09354 | 10.6813 | 0.28962205 |
|  | *Gm8817* | 2.94197 | 14.7734 | 0.19913967 |
|  | *Clpp* | 0.607847 | 20.5193 | 0.029623184 |
