# Supplementary material for: Mitochondrial unfolded protein response gene Clpp is required to maintain ovarian follicular reserve during aging, for oocyte competence, and development of pre‐implantation embryos
Source: Aging Cell. 2018 May 30;17(4):e12784. doi: 10.1111/acel.12784 (PMC6052477; doi:10.1111/acel.12784)
Supplement: Supplementary file 11 [file ACEL-17-na-s011.docx]

**EXPERIMENTAL PROCEDURES**

**Mouse breeding and genotyping**

*Clpp*^+/-^ male and female mice from the founder line IST13563G11 (line G) in the inbred C57BL/6J genetic background were acquired from Georg Auburger, PhD (Goethe University Medical School, Frankfort am Main, Germany) (Gispert *et al.* 2013), and bred to obtain *Clpp*^-/-^ mice. Animals were housed under a 12-hour light-dark cycle and food and water were provided ad libitum. Mice were bred and maintained according to Yale University animal research requirements, using protocols approved by the Institutional Animal Care and Use Committee (protocol #2011-11207). Genotyping was carried out using the method previously described (Gispert *et al.* 2013).

**Histomorphometric analysis of folliculogenesis in ovaries**

For hematoxylin and eosin (HE) staining, ovaries from 3-month-old *Clpp*^+/+^ and *Clpp*^-/-^ mice were fixed in 4% (w/v) paraformaldehyde in Dulbecco's phosphate buffered saline (DPBS; Sigma) at room temperature overnight, and stored at 4ºC in fresh 70% ethanol until further use. Ovaries were then dehydrated, embedded in paraffin, and 5 μm serial sections were stained with HE using standard protocol (Myers *et al.* 2004). Every 5th section was assessed, and the total number of follicles for each ovary was determined by counting the follicles containing oocytes with a visible nucleus. The follicle density was calculated by dividing total follicle number by the total section area. Primordial, primary, secondary, antral and atretic follicles were classified as described previously (Guzeloglu-Kayisli *et al.* 2012). Briefly, primordial follicles were defined as an oocyte surrounded by a layer of squamous granulosa cells. Primary follicles possessed an oocyte surrounded by a single layer of cuboidal granulosa cells. Secondary follicles were surrounded by two or more layers of cuboidal granulosa cells with no visible antrum. Antral follicles contained four or more layers of granulosa cells with a clearly defined single antral space.

**Oocyte and embryo collection**

Mouse oocytes and embryos were collected using standard protocols (Seli *et al.* 2005). Briefly, mature female mice were superovulated by intra-peritoneal injection of 5 IU of pregnant mare serum gonadotropin (PMSG; Sigma, St. Louis, MO) to stimulate follicle development. To collect oocytes arrested at GV (germinal vesicle) stage, mice were euthanized 44 h later by CO2 inhalation, the ovaries were removed, and GV oocytes were isolated by puncturing the ovaries with a 26-1/2 G needle under the dissecting microscope (Olympus SZH-ILLK). To obtain mature oocytes or embryos, an additional injection of 5 IU of human chorionic gonadotrophin (hCG; Sigma) to induce oocyte maturation and ovulation was given 48 h after the PMSG injection. Unfertilized oocytes at metaphase of the second meiotic division (MII) were collected from oviducts 14 h after the hCG injection. To obtain fertilized embryos, females were placed individually with 12-week-old WT males immediately after the hCG injection. The following morning, the effectiveness of mating was confirmed by the presence of a vaginal plug. Two-cell embryos were collected 44 h after hCG injection from the oviducts into KSOM medium (Millipore, Billerica, MA). Removal of the cumulus cells was achieved in KSOM medium containing 1 mg/ml hyaluronidase (Sigma). Blastocysts were collected 92 h after hCG injection from uterus into M2 medium (Millipore).

***In vitro* maturation (IVM) of oocytes**

GV stage oocytes were collected in a-minimum essential medium (MEMa; Life Technologies, Grand Island, NY) supplemented with 20 mM Hepes, 75μg/ml penicillin G (Sigma), 50μg/ml streptomycin sulfate (Sigma), 0.1% polyvinyl alcohol (PVA; Sigma), and 10μM milrinone (Sigma) to prevent meiotic resumption. For IVM, GV oocytes were transferred to MEMa supplemented with 25 mM NaHCO3, 75 μg/ml penicillin G, 50μg/ml streptomycin sulfate, 5% fetal bovine serum (FBS; product no. 12000-022; Life Technologies) and incubated in a humidified atmosphere at 37ºC with 5% CO2 and 95% air. Oocytes were assessed for 18 h in culture for GV breakdown (GVBD).

**Immunostaining of oocyte spindles**

Oocytes were fixed in 4% (w/v) paraformaldehyde in DPBS (pH 7.4) for 30 min, permeabilized in 0.5% Triton X-100 for 5 min. Then, oocytes were incubated with 2μg/ml anti-α-Tubulin (Cat#16-232, Millipore, Billerica, MA) for 1 h, washed three times for 5 min in DPBS and stained with 4’,6-diamidino-2-phenylindole (DAPI, Life Technologies, ThermoFisher Scientific, Waltham, MA) prior to being examined using Leica SP5 spectral scanning confocal microscope (Leica Microsystem, Buffalo Grove, IL) using excitation at 488 nm and emission at 530 nm (tubulin) and excitation at 350 nm and emission at 470 nm (DAPI).

**Electron microscopic (EM) analysis of mitochondrial dynamics**

3 WT and 3 KO female mice were deeply anesthetized 44h after PMSG injection and perfused with saline containing heparin followed by fixative solution (Paraformaldehyde 4%, gluteraldehyde 0.1%, picric acid 15%, in phosphate buffer (PB) 0.1 M, pH = 7.4). Both ovaries were removed and fixed overnight at 4ºC with the same fixative without gluteraldehyde and then dehydrated using ethanol gradient. Ovaries were then embedded in Durcupan, cut in an ultramicrotome and collected in grids for ultrastructural analysis with Tecnai 12 Biotwin electron microscope. GV oocytes were imaged at 1900x magnification. Image J software was used to outline each individual mitochondrion, nucleus and cell membrane in oocytes. Mitochondria cross-sectional area was used as the measurement of mitochondria size. Mitochondrion aspect ratio was used as an index of mitochondrion shape (AR, major axis divided by minor axis, minimum value is 1.0).

**Quantification of mtDNA copy number in oocytes**

To quantify mtDNA levels in GV and MII oocytes, *Cox3* fragment was amplified using the primers in Supplementary Table S1 and subcloned into pCR™2.1-TOPO® - cloning vector (Invitrogen) as previously described (Babayev *et al.* 2016). One Shot TOP10 Chemically Competent E. coli were transformed and grown overnight at 37ºC. Recombinant plasmids were purified using Qiagen plasmid isolation kit and the inserted mtDNA fragment was confirmed by DNA sequence analysis. Plasmid DNA was quantified using NanoDrop 2000 spectrophotometer (Thermo Scientific). A standard curve from 10^8^ to 10^1^ plasmid molecules was generated by serial 10-fold dilutions. Individual GV or MII oocytes were lysed in 10 μl lysis solution containing 125 μg/ml Proteinase K and 17 μM SDS in sterile water by incubating at 55ºC for 2 h. Then Proteinase K was inactivated by heating the lysis mix at 95ºC for 10 min and the mix was used directly for downstream PCR. Reactions were performed in triplicates. Each 10 μl reaction contained 5 μl of SYBR Green supermix (Bio-Rad Laboratories, Hercules, CA), approximately 0.3 μM of each primer, and 1/3 of total oocyte DNA. Oocyte mtDNA copy numbers were extrapolated from the standard curve.

**Determination of ROS levels**

6-carboxy-2', 7’-dichlorodihydrofluorescein diacetate (carboxy-H2DCFDA) (Life Technologies, ThermoFisher Scientific, cat # c-400) was used to assess ROS levels in mouse oocytes (Takahashi *et al.* 2003). We induced ROS generation by exposing GV oocytes to 20mM H2O2 for 5 min and then incubated these oocytes with 30 µM H2DCFDA in HEPES buffered MEMα for 20 min. Oocytes were washed 3 times in H2DCFDA-free media and images were captured on Leica SP5 spectral scanning confocal microscope. Image J software was used to quantify fluorescence.

**Analysis of mitochondrial membrane potential**

To measure mitochondrial membrane potential, oocytes were incubated with the mitochondrial membrane dye JC-1 (Invitrogen) at 2μg/ml for 30 min at 37ºC in the dark. Oocytes were then washed 3 times in a JC-1-free media and imaged immediately in both green and red fluorescence channels using excitation at 488 nm and emission at 530 nm (green) and excitation at 568 nm and emission at 590 nm (red) with the Leica SP5 spectral scanning confocal microscope at the same magnification and gain settings throughout experiments. Image J software was used to quantify intensity.

**Quantitation of ATP**

ATP content of individual oocytes was determined using the ATP bioluminescent somatic cell assay kit (Sigma). Oocytes were collected, lysed, and stored in 100μl somatic cell ATP releasing reagent at -80ºC. ATP Assay Mix Working Solution (100 μl, 1:25 diluted from ATP Assay Mix Stock Solution) was then added individually to 96-well plate and kept at room temperature for 3–5 minutes to allow endogenous ATP hydrolysis. To a separate vial containing 100μl of 1X ice-cold somatic cell ATP-releasing reagent, 50μl of ultrapure water and 50μl of samples to be assayed or standards were added and swirled briskly; 100μl of the mix was transferred individually to 96-well plate and the amount of light emitted was measured immediately with Dynex MLX microliter plate luminometer (Dynex Technologies, Chantilly, VA). The background luminescence was subtracted from all readings. ATP in single oocyte samples was calculated by comparison to a standard curve generated over the range 2.5–500 fmol/100μl.

**Quantitative reverse-transcription polymerase chain reaction (qRT-PCR)**

Total RNA was obtained from 20 oocytes per mice using RNAqueous Microkit (Ambion, Austin, TX) and was treated for genomic DNA contamination using DNase I (Ambion). Reverse transcription was performed using the RETROscript kit (Ambion) in two steps: first, template RNA and oligo(dT) primers were incubated at 85ºC for 3 min to eliminate any secondary structures, and then the buffer and enzyme were added and the reaction was carried out at 42ºC for 1 h. qRT-PCR was carried out on an iCycler (Bio-Rad Laboratories). cDNA was prepared as described above, and assayed in triplicate. Each experiment was repeated at least three times using individual animals from each genotype. Each 10-µl reaction contained 5µl of SYBR Green supermix (Bio-Rad Laboratories), 3µl of H_2_O, 0.5µl of each primer, and 1µl of cDNA. TaqMan Gene expression assays (Life Technologies) were also used following manufacturer’s instructions. Briefly, each 20 µl reaction contained 1 µl of 20X TaqMan gene expression assay, 10 µl of 2X TaqMan Gene expression master mix, 4 µl of cDNA template and 5 µl of H2O. The 2-^∆∆^CT (cycle threshold) method was used to calculate relative expression levels after normalization to β-actin or *Gapdh* levels. The primers used for real-time PCR reactions are given in Supplementary Table 1 (Table S1).

**RNA sequencing and data analysis**

cDNA synthesis and amplification was performed using 5 GV oocytes per sample from both 3 and 6 months *ClpP*^+/+^ and *ClpP*^-/-^ mice and Smart-Seq2 protocol as previously described (Ramsköld *et al.* 2012; Picelli *et al.* 2013). Briefly, poly(A) RNAs were reverse-transcribed through tailed oligo(dT) priming using the CDS primer (5′-AAGCAGTGGTATCAACGCAGAGTACT(30)VN-3′, where V represents A, C or G) directly in whole cells lysate in 0.2% Triton X-100 and 2U/ul of RNase inhibitors (Invitrogen) in RNase free water. First-strand cDNA generation was carried out with the addition of 5X SuperScript II first-strand buffer, dithiothreitol (100ml), RNAse inhibitor, SuperScript II reverse transcriptase (100 U) (Invitrogen), and TSO primers (5’-AAGCAGTGGTATCAACGCAGAGTACATrGrG+G-3’, where r indicate ribonucleotide bases). The cDNA was then amplified using KAPA HIFI hotStart ReadyMix (2x) (KAPA Biosystems) with PCR primers (5′-AAGCAGTGGTATCAACGCAGAGT-3′). The quality of amplified cDNA was measured by Qubit 3.0 (Life Technologies) and High Sensitivity kits on Tapestation 2200 (Agilent Technologies, Santa Clara, CA).

Sequencing libraries were constructed using the amplified cDNA and Nextera® XT DNA Library Preparation Kit (Illumina, San Diego, CA) and indexed using Nextera® XT Index Kit (Illumina). Library quality was confirmed using DNA 1000 kit on Tapestation 2200 (Aglilent Technologies), and the libraries were then sequenced on Illumina’s Hiseq 2500 platform with 75bp pair-end reads. In total, we analyzed 6 samples and obtained about 212 million reads. The raw FASTQ files and normalized gene expression levels are available at Gene Expression Omnibus (GEO) (www.ncbi.nlm.nih.gov/geo) under the accession number GSE102269.

Multiplexed sequencing reads that passed filters were trimmed as previously described (Jiang *et al.* 2014). Briefly, sequencing adapters were trimmed using Cutadapt and low quality reads were pre-filtered by FASTX-Toolkit before mapping. The quality of reads after filtering was examined using fastQC, followed by alignment to the mouse genome (GRCm38/mm10) by Tophat (v2.0.10) using SAMtools (v0.1.18) and Bowtie (v2.1.0) with default parameters (Trapnell *et al.* 2009; Trapnell *et al.* 2012). Individual mapped reads were fed to Cufflinks (v1.2.1) (Trapnell *et al.* 2012) to construct transcriptome models and any novel genes and transcripts that did not fit the supplied gene models were also assembled. Cuffmerge (Trapnell *et al.* 2012) was used to converge individual transcriptome to produce a master gene model. Then Cufflinks was run to calculate Fragments Per Kilobase of exon model per Million mapped fragments (FPKM) using RefSeq genes as reference (Trapnell *et al.* 2012). We filtered out genes with FPKM < 0.1 in both groups. A matrix of Pearson correlation coefficient was created using R Package. Differential gene expression analysis was performed using Cuffdiff (Trapnell *et al.* 2009) with default parameters. The multiple testing errors were corrected by the false discovery rate (FDR) of < 0.05. Expression pattern clusters were generated by the unsupervised hierarchical clustering analysis and K-means clustering algorithm using R. DAVID and IPA were used to reveal the Gene Ontology (GO) and pathways.

**Western blotting**

Protein was extracted from ovaries using RIPA lysis buffer containing a protease inhibitor cocktail (Thermo Scientific) with PhosSTOP (Roche, Basel, Switzerland). Equal amounts of protein per sample were separated by 10% sodium dodecyl sulfate-PAGE (Bio-Rad Laboratories) and transferred electrophoretically to a nitrocellulose membrane (Bio-Rad Laboratories) at 100 V for 2 h or 25V overnight. The membrane was blocked with 5% BSA in Tris-buffered saline with Tween-20 for 1 h at room temperature and blotted with antibodies against, pS6 (Cat. # 4856, Cell Signaling, 1:1000), S6 (Cat. # 2217, Cell Signaling, 1:1000), pS6K (Cat. # 9234, Cell Signaling, 1:1000), S6K (Cat. # 2708, Cell Signaling, 1:1000), p4EBP1 (Cat. # 2855, Cell Signaling, 1:1000), 4EBP1 (Cat. # 9452, Cell Signaling, 1:1000), pAKT473 (Cat. # 4060, Cell Signaling, 1:1000), AKT (Cat. # 2965, Cell Signaling, 1:1000), pmTOR2481 (Cat. # 2974, Cell Signaling, 1:1000), mTOR (Cat. # 2983, Cell Signaling, 1:1000), β-ACTIN (Cat. # A5136, Sigma-Aldrich, 1:5000) and HSP90 (Cat. # SAB4300541, Sigma-Aldrich, 1:5000) at 4°C overnight. The membrane was then washed 3 times in TBS-T and incubated with horseradish peroxidase-conjugated secondary antibody (1:5000; Chemicon) diluted in TBS-T, for 1 h at room temperature. Bound antibody was detected with Western Lightning Plus-ECL (PerkinElmer, Shelton, CT). Images of blot signals on HyBlot ES® Autoradiography Film (E3012) were scanned on an Epson 4490 scanner, and viewed using Image J software (National Institutes of Health) for data analysis. Levels of p-S6, p-S6K, p-4EBP1,p-AKT473 and p-mTOR2481 were normalized to total S6, total S6K, total 4EBP1,total AKT473 and total mTOR, respectively.

**Immunofluorescent staining**

Ovaries were embedded in paraffin and sectioned at a thickness of 5 μm. Slides were dewaxed in xylene, boiled for 30 min in citrate buffer for antigen retrieval, and rehydrated. After being washed three times with PBS, sections were incubated with antibodies against phosphorylated S6 (pS6) (catalog no. 4856, Cell Signaling, 1:100) and pAKT473 (catalog no. 4060, Cell Signaling, 1:100) diluted in blocking solution (horse serum and 10% BSA in PBS) overnight at 4°C in a humidified chamber. Sections were then washed three times with Tris-buffered saline, incubated with Alexa fluor 594-conjugated secondary antibodies diluted 1:1,000 in blocking solution for 1 h at room temperature, washed again three times, and mounted on slides with Pro-Long Gold Mounting Reagent with DAPI (Life Technologies). Ovary sections were prepared as mentioned above. TUNEL assay (Sigma-Aldrich ) was applied according to the manual. Ki67 (Cell Signaling) detection was applied to assess cell proliferation.

**Metabolic Analysis**

Sera were sent to University of Cincinnati Mouse Metabolic and Phenotyping Center. Lipid profiles were assessed in four different parameters: triglycerides, total cholesterol, phospholipids and non-esterified fatty acids (NEFA). Reactions were run in microtiter plates and analyzed on a plate reader. Blood glucose levels were measured with a calorimetric assay in microtiter plates.

**AMH and Estradiol test**

For serum AMH and estradiol analyses, samples were sent to The University of Virginia Center for Research in Reproduction Ligand Assay and Analysis Core. ANSH ELISA kit was used for AMH study, and Calbiotech ELISA kits were used for estradiol study.

**Rapamycin rescue treatment**

Rapamycin rescue experiments were performed *in vivo* and *in vitro*. In vivo, 2mg/kg rapamycin (Millipore) was injected intraperitoneally once a day for 14 days prior to experiments. In vitro, 1nM rapamycin was added to the IVM culture medium. GVBD was followed under IVM conditions up to 18 h. Spindle morphology was assessed in MII oocytes both *in vivo* and *in vitro*.

Babayev E, Wang T, Lowther K, Horvath T, Taylor HS, Seli E (2016). Aging is associated with changes in mitochondrial dynamics, function and mtDNA quantity. . *Maturitas*. **Epub June 23**.

Gispert S, Parganlija D, Klinkenberg M, Dröse S, Wittig I, Mittelbronn M, Grzmil P, Koob S, Hamann A, Walter M, et al. (2013). Loss of mitochondrial peptidase Clpp leads to infertility, hearing loss plus growth retardation via accumulation of CLPX, mtDNA and inflammatory factors. *Hum Mol Genet*. **22**, 4871-4887.

Guzeloglu-Kayisli O, Lalioti MD, Aydiner F, Sasson I, Ilbay O, Sakkas D, Lowther KM, Mehlmann LM, Seli E (2012). Embryonic poly(A) binding protein (EPAB) is required for oocyte maturation and female fertility in mice. *Biochem J*. **446**, 47-58.

Jiang Z, Sun J, Dong H, Luo O, Zheng X, Obergfell C, Tang Y, Bi J, O'Neill R, Ruan Y, et al. (2014). Transcriptional profiles of bovine in vivo pre-implantation development. *BMC Genomics*. **15**, 756.

Myers M, Britt KL, Wreford NG, Ebling FJ, Kerr JB (2004). Methods for quantifying follicular numbers within the mouse ovary. *Reproduction*. **127**, 569-580.

Picelli S, Björklund ÅK, Faridani OR, Sagasser S, Winberg G, Sandberg R (2013). Smart-seq2 for sensitive full-length transcriptome profiling in single cells. *Nat Methods*. **10**, 1096-1098.

Ramsköld D, Luo S, Wang YC, Li R, Deng Q, Faridani OR, Daniels GA, Khrebtukova I, Loring JF, Laurent LC, et al. (2012). Full-length mRNA-Seq from single-cell levels of RNA and individual circulating tumor cells. *Nat Biotechnol*. **30**, 777-782.

Seli E, Lalioti MD, Flaherty SM, Sakkas D, Terzi N, Steitz JA (2005). An embryonic poly(A)-binding protein (ePAB) is expressed in mouse oocytes and early preimplantation embryos. *Proc Natl Acad Sci USA*. **102**, 367-372.

Takahashi T, Takahashi E, Igarashi H, Tezuka N, Kurachi H (2003). Impact of oxidative stress in aged mouse oocytes on calcium oscillations at fertilization. *Mol Reprod Dev*. **66**, 143-152.

Trapnell C, Pachter L, Salzberg SL (2009). TopHat: discovering splice junctions with RNA-Seq. . *Bioinformatics*. **25**, 1105-1111.

Trapnell C, Roberts A, Goff L, Pertea G, Kim D, Kelley DR, Pimentel H, Salzberg SL, Rinn JL, Pachter L (2012). Differential gene and transcript expression analysis of RNA-seq experiments with TopHat and Cufflinks. *Nat Protoc*. **7**, 562-578.
